# Supplementary material for: IS-Seq: a bioinformatics pipeline for integration sites analysis with comprehensive abundance quantification methods
Source: BMC Bioinformatics. 2023 Jul 18;24:286. doi: 10.1186/s12859-023-05390-1 (PMC10354991; doi:10.1186/s12859-023-05390-1)
Supplement: Supplementary file 1 — Additional file 1: Table S1. Oligos used for insertion sites retrieval. Table S2. LTR fusion primers used for Illumina sequencing library preparation. Table S3. LC fusion primers used for Illumina sequencing library preparation. Table S4. Comparison of expected and observed result from IS-Seq run on 4 in silico simulations as described in the manuscript text. Table S5. Correlation coefficientbetween IS relative abundance calculated with INSPIIRED versus IS-Seq on single cell clones with known copy number. Table S6. Mean square errorcalculated between the shared IS resulting from each IS-Seq pipeline iterationand the expected relative abundance of the most abundant IS detected for each clone. The average MSE for all the results of each pipeline iteration is reported in the last row of the table. Table S7. Mean square errorcalculated between the shared IS resulting from each IS-Seq pipeline iteration and the expected relative abundance of the serial dilutions of CL6 into a bulk HL60 transduced polyclonal population. The average MSE for all the results of each pipeline iteration is reported in the last row of the table. Table S8. Number of R2 reads, total UMIs and Unique UMIs derived from the 3 datasets object of this study. Table S9. Mean square errorcalculated on a 100 IS simulated dataset with serial IS dilutions using different levels of PCR duplicates and UMI diversity. [file 12859_2023_5390_MOESM1_ESM.pptx]

## Slide 1
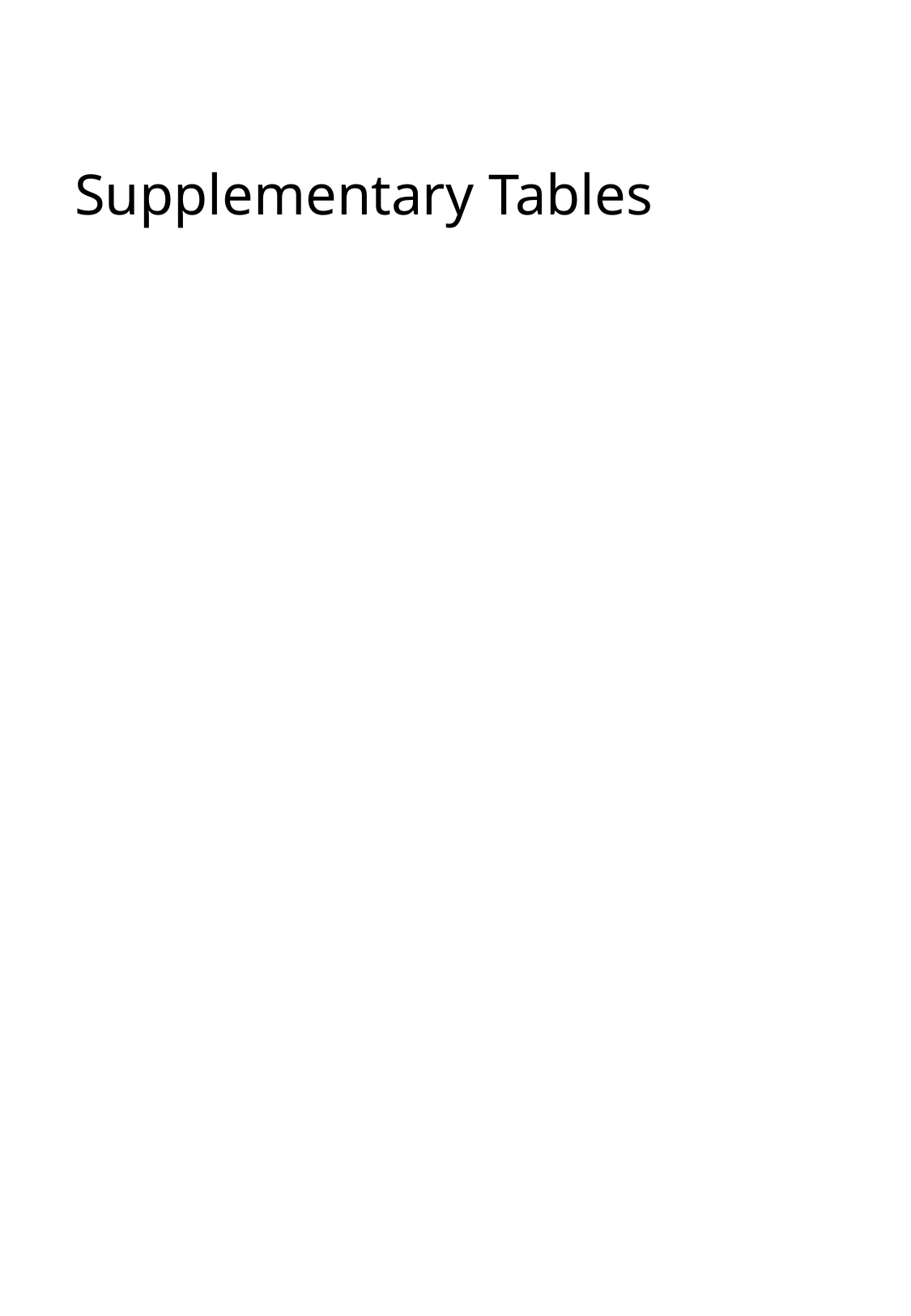

# Supplementary Tables

## Slide 2
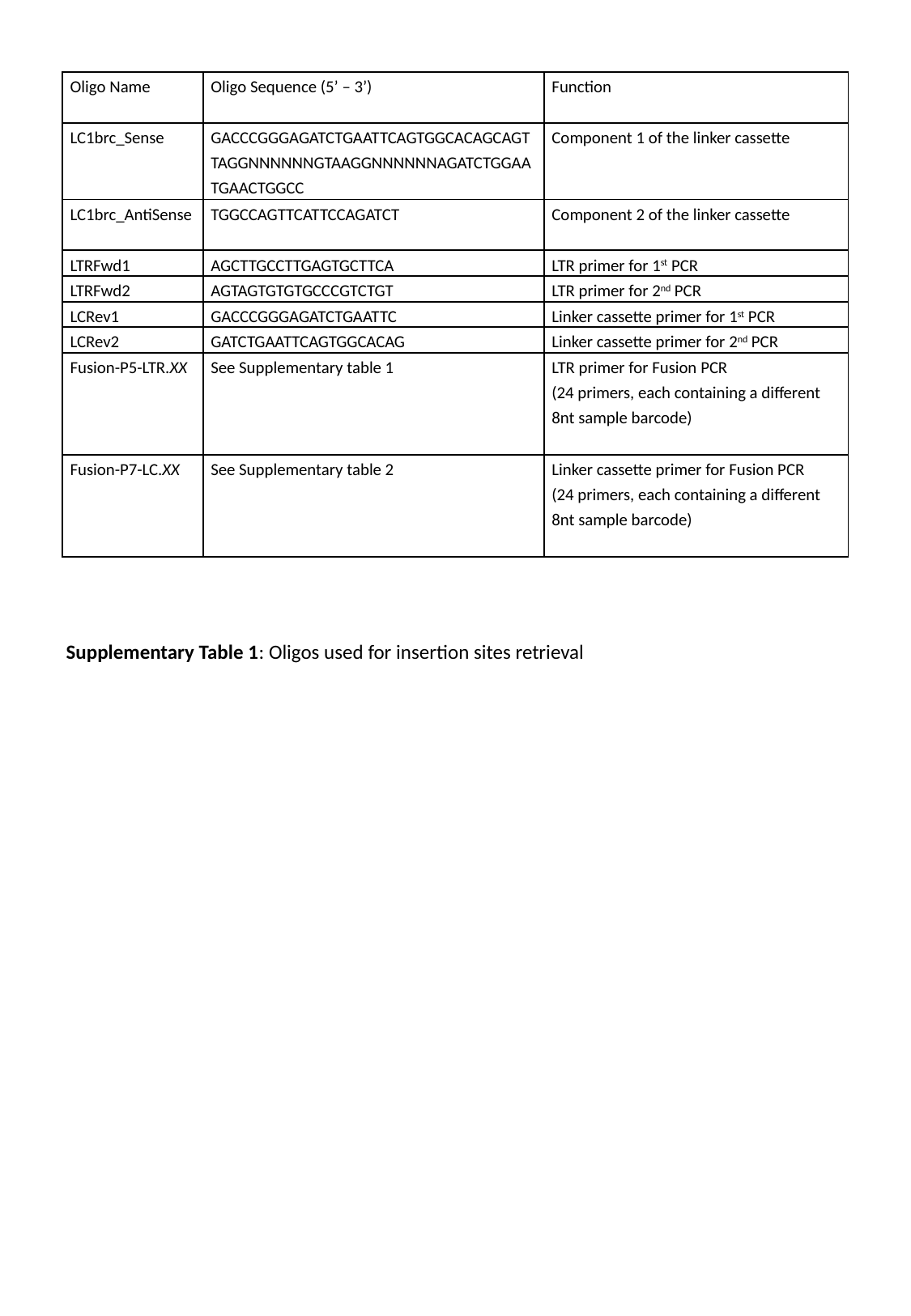

| Oligo Name | Oligo Sequence (5’ – 3’) | Function |
| --- | --- | --- |
| LC1brc\_Sense | GACCCGGGAGATCTGAATTCAGTGGCACAGCAGTTAGGNNNNNNGTAAGGNNNNNNAGATCTGGAATGAACTGGCC | Component 1 of the linker cassette |
| LC1brc\_AntiSense | TGGCCAGTTCATTCCAGATCT | Component 2 of the linker cassette |
| LTRFwd1 | AGCTTGCCTTGAGTGCTTCA | LTR primer for 1st PCR |
| LTRFwd2 | AGTAGTGTGTGCCCGTCTGT | LTR primer for 2nd PCR |
| LCRev1 | GACCCGGGAGATCTGAATTC | Linker cassette primer for 1st PCR |
| LCRev2 | GATCTGAATTCAGTGGCACAG | Linker cassette primer for 2nd PCR |
| Fusion-P5-LTR.XX | See Supplementary table 1 | LTR primer for Fusion PCR (24 primers, each containing a different 8nt sample barcode) |
| Fusion-P7-LC.XX | See Supplementary table 2 | Linker cassette primer for Fusion PCR (24 primers, each containing a different 8nt sample barcode) |
Supplementary Table 1: Oligos used for insertion sites retrieval

## Slide 3
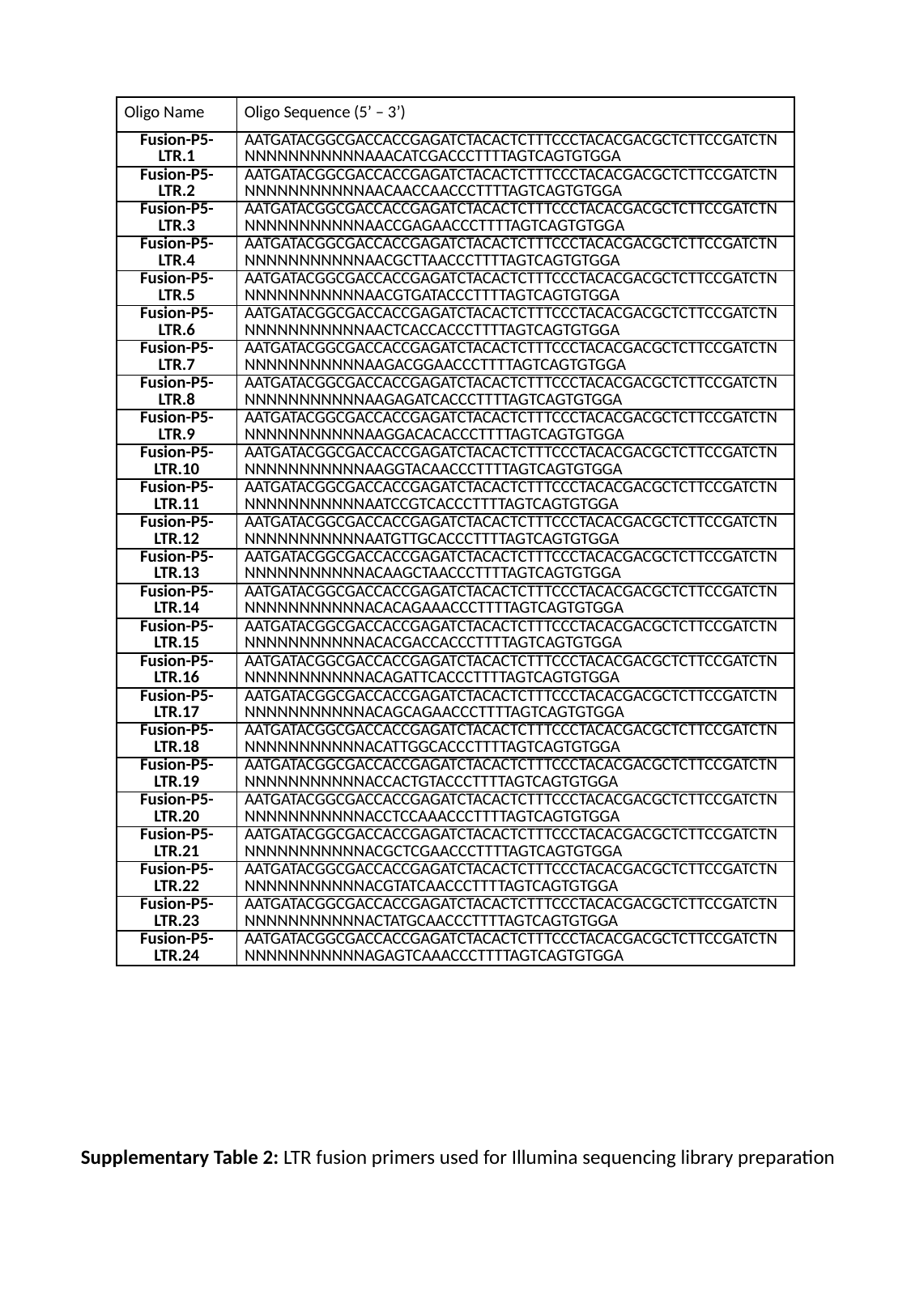

| Oligo Name | Oligo Sequence (5’ – 3’) |
| --- | --- |
| Fusion-P5-LTR.1 | AATGATACGGCGACCACCGAGATCTACACTCTTTCCCTACACGACGCTCTTCCGATCTNNNNNNNNNNNNAAACATCGACCCTTTTAGTCAGTGTGGA |
| Fusion-P5-LTR.2 | AATGATACGGCGACCACCGAGATCTACACTCTTTCCCTACACGACGCTCTTCCGATCTNNNNNNNNNNNNAACAACCAACCCTTTTAGTCAGTGTGGA |
| Fusion-P5-LTR.3 | AATGATACGGCGACCACCGAGATCTACACTCTTTCCCTACACGACGCTCTTCCGATCTNNNNNNNNNNNNAACCGAGAACCCTTTTAGTCAGTGTGGA |
| Fusion-P5-LTR.4 | AATGATACGGCGACCACCGAGATCTACACTCTTTCCCTACACGACGCTCTTCCGATCTNNNNNNNNNNNNAACGCTTAACCCTTTTAGTCAGTGTGGA |
| Fusion-P5-LTR.5 | AATGATACGGCGACCACCGAGATCTACACTCTTTCCCTACACGACGCTCTTCCGATCTNNNNNNNNNNNNAACGTGATACCCTTTTAGTCAGTGTGGA |
| Fusion-P5-LTR.6 | AATGATACGGCGACCACCGAGATCTACACTCTTTCCCTACACGACGCTCTTCCGATCTNNNNNNNNNNNNAACTCACCACCCTTTTAGTCAGTGTGGA |
| Fusion-P5-LTR.7 | AATGATACGGCGACCACCGAGATCTACACTCTTTCCCTACACGACGCTCTTCCGATCTNNNNNNNNNNNNAAGACGGAACCCTTTTAGTCAGTGTGGA |
| Fusion-P5-LTR.8 | AATGATACGGCGACCACCGAGATCTACACTCTTTCCCTACACGACGCTCTTCCGATCTNNNNNNNNNNNNAAGAGATCACCCTTTTAGTCAGTGTGGA |
| Fusion-P5-LTR.9 | AATGATACGGCGACCACCGAGATCTACACTCTTTCCCTACACGACGCTCTTCCGATCTNNNNNNNNNNNNAAGGACACACCCTTTTAGTCAGTGTGGA |
| Fusion-P5-LTR.10 | AATGATACGGCGACCACCGAGATCTACACTCTTTCCCTACACGACGCTCTTCCGATCTNNNNNNNNNNNNAAGGTACAACCCTTTTAGTCAGTGTGGA |
| Fusion-P5-LTR.11 | AATGATACGGCGACCACCGAGATCTACACTCTTTCCCTACACGACGCTCTTCCGATCTNNNNNNNNNNNNAATCCGTCACCCTTTTAGTCAGTGTGGA |
| Fusion-P5-LTR.12 | AATGATACGGCGACCACCGAGATCTACACTCTTTCCCTACACGACGCTCTTCCGATCTNNNNNNNNNNNNAATGTTGCACCCTTTTAGTCAGTGTGGA |
| Fusion-P5-LTR.13 | AATGATACGGCGACCACCGAGATCTACACTCTTTCCCTACACGACGCTCTTCCGATCTNNNNNNNNNNNNACAAGCTAACCCTTTTAGTCAGTGTGGA |
| Fusion-P5-LTR.14 | AATGATACGGCGACCACCGAGATCTACACTCTTTCCCTACACGACGCTCTTCCGATCTNNNNNNNNNNNNACACAGAAACCCTTTTAGTCAGTGTGGA |
| Fusion-P5-LTR.15 | AATGATACGGCGACCACCGAGATCTACACTCTTTCCCTACACGACGCTCTTCCGATCTNNNNNNNNNNNNACACGACCACCCTTTTAGTCAGTGTGGA |
| Fusion-P5-LTR.16 | AATGATACGGCGACCACCGAGATCTACACTCTTTCCCTACACGACGCTCTTCCGATCTNNNNNNNNNNNNACAGATTCACCCTTTTAGTCAGTGTGGA |
| Fusion-P5-LTR.17 | AATGATACGGCGACCACCGAGATCTACACTCTTTCCCTACACGACGCTCTTCCGATCTNNNNNNNNNNNNACAGCAGAACCCTTTTAGTCAGTGTGGA |
| Fusion-P5-LTR.18 | AATGATACGGCGACCACCGAGATCTACACTCTTTCCCTACACGACGCTCTTCCGATCTNNNNNNNNNNNNACATTGGCACCCTTTTAGTCAGTGTGGA |
| Fusion-P5-LTR.19 | AATGATACGGCGACCACCGAGATCTACACTCTTTCCCTACACGACGCTCTTCCGATCTNNNNNNNNNNNNACCACTGTACCCTTTTAGTCAGTGTGGA |
| Fusion-P5-LTR.20 | AATGATACGGCGACCACCGAGATCTACACTCTTTCCCTACACGACGCTCTTCCGATCTNNNNNNNNNNNNACCTCCAAACCCTTTTAGTCAGTGTGGA |
| Fusion-P5-LTR.21 | AATGATACGGCGACCACCGAGATCTACACTCTTTCCCTACACGACGCTCTTCCGATCTNNNNNNNNNNNNACGCTCGAACCCTTTTAGTCAGTGTGGA |
| Fusion-P5-LTR.22 | AATGATACGGCGACCACCGAGATCTACACTCTTTCCCTACACGACGCTCTTCCGATCTNNNNNNNNNNNNACGTATCAACCCTTTTAGTCAGTGTGGA |
| Fusion-P5-LTR.23 | AATGATACGGCGACCACCGAGATCTACACTCTTTCCCTACACGACGCTCTTCCGATCTNNNNNNNNNNNNACTATGCAACCCTTTTAGTCAGTGTGGA |
| Fusion-P5-LTR.24 | AATGATACGGCGACCACCGAGATCTACACTCTTTCCCTACACGACGCTCTTCCGATCTNNNNNNNNNNNNAGAGTCAAACCCTTTTAGTCAGTGTGGA |
Supplementary Table 2: LTR fusion primers used for Illumina sequencing library preparation

## Slide 4
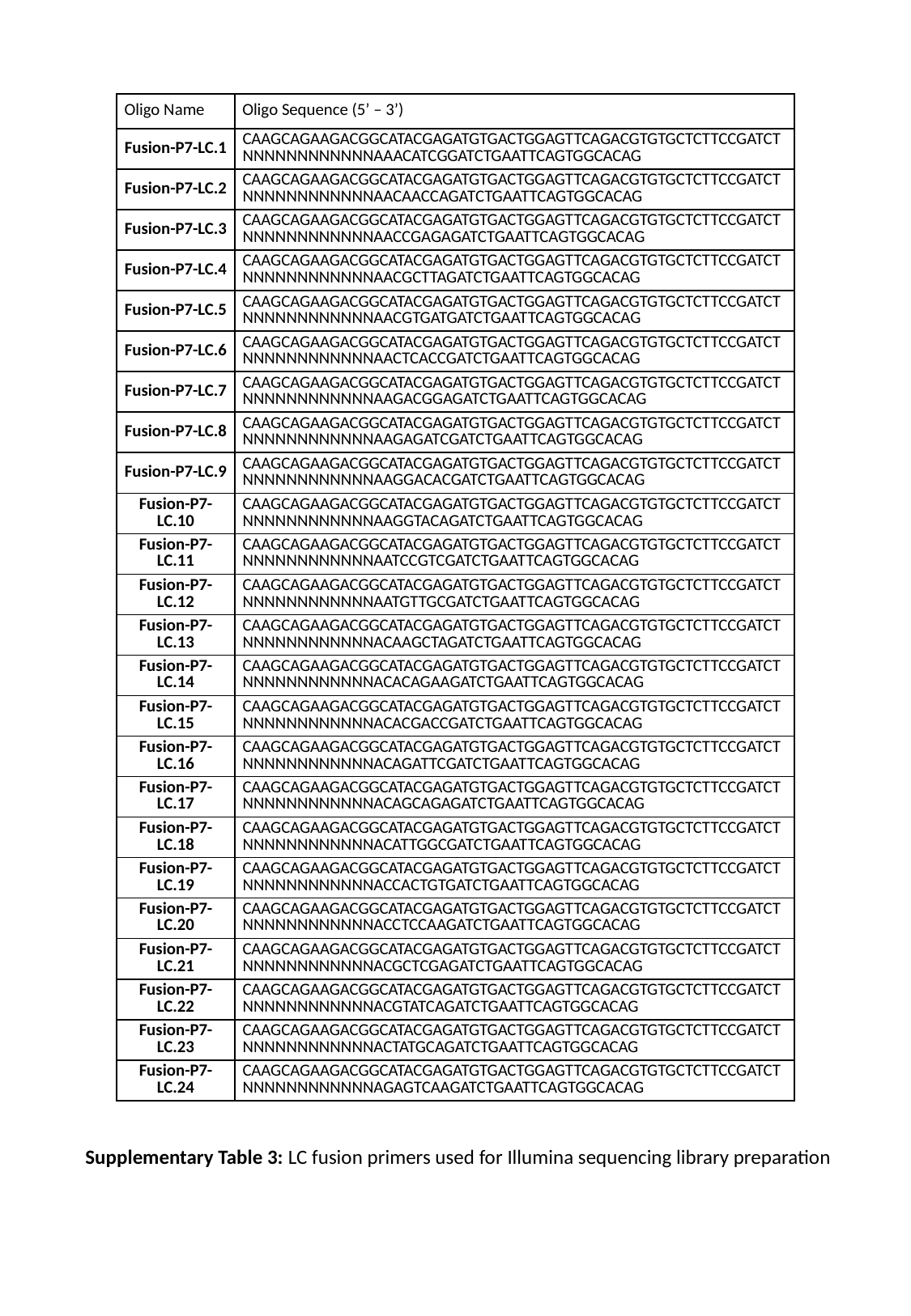

| Oligo Name | Oligo Sequence (5’ – 3’) |
| --- | --- |
| Fusion-P7-LC.1 | CAAGCAGAAGACGGCATACGAGATGTGACTGGAGTTCAGACGTGTGCTCTTCCGATCTNNNNNNNNNNNNAAACATCGGATCTGAATTCAGTGGCACAG |
| Fusion-P7-LC.2 | CAAGCAGAAGACGGCATACGAGATGTGACTGGAGTTCAGACGTGTGCTCTTCCGATCTNNNNNNNNNNNNAACAACCAGATCTGAATTCAGTGGCACAG |
| Fusion-P7-LC.3 | CAAGCAGAAGACGGCATACGAGATGTGACTGGAGTTCAGACGTGTGCTCTTCCGATCTNNNNNNNNNNNNAACCGAGAGATCTGAATTCAGTGGCACAG |
| Fusion-P7-LC.4 | CAAGCAGAAGACGGCATACGAGATGTGACTGGAGTTCAGACGTGTGCTCTTCCGATCTNNNNNNNNNNNNAACGCTTAGATCTGAATTCAGTGGCACAG |
| Fusion-P7-LC.5 | CAAGCAGAAGACGGCATACGAGATGTGACTGGAGTTCAGACGTGTGCTCTTCCGATCTNNNNNNNNNNNNAACGTGATGATCTGAATTCAGTGGCACAG |
| Fusion-P7-LC.6 | CAAGCAGAAGACGGCATACGAGATGTGACTGGAGTTCAGACGTGTGCTCTTCCGATCTNNNNNNNNNNNNAACTCACCGATCTGAATTCAGTGGCACAG |
| Fusion-P7-LC.7 | CAAGCAGAAGACGGCATACGAGATGTGACTGGAGTTCAGACGTGTGCTCTTCCGATCTNNNNNNNNNNNNAAGACGGAGATCTGAATTCAGTGGCACAG |
| Fusion-P7-LC.8 | CAAGCAGAAGACGGCATACGAGATGTGACTGGAGTTCAGACGTGTGCTCTTCCGATCTNNNNNNNNNNNNAAGAGATCGATCTGAATTCAGTGGCACAG |
| Fusion-P7-LC.9 | CAAGCAGAAGACGGCATACGAGATGTGACTGGAGTTCAGACGTGTGCTCTTCCGATCTNNNNNNNNNNNNAAGGACACGATCTGAATTCAGTGGCACAG |
| Fusion-P7-LC.10 | CAAGCAGAAGACGGCATACGAGATGTGACTGGAGTTCAGACGTGTGCTCTTCCGATCTNNNNNNNNNNNNAAGGTACAGATCTGAATTCAGTGGCACAG |
| Fusion-P7-LC.11 | CAAGCAGAAGACGGCATACGAGATGTGACTGGAGTTCAGACGTGTGCTCTTCCGATCTNNNNNNNNNNNNAATCCGTCGATCTGAATTCAGTGGCACAG |
| Fusion-P7-LC.12 | CAAGCAGAAGACGGCATACGAGATGTGACTGGAGTTCAGACGTGTGCTCTTCCGATCTNNNNNNNNNNNNAATGTTGCGATCTGAATTCAGTGGCACAG |
| Fusion-P7-LC.13 | CAAGCAGAAGACGGCATACGAGATGTGACTGGAGTTCAGACGTGTGCTCTTCCGATCTNNNNNNNNNNNNACAAGCTAGATCTGAATTCAGTGGCACAG |
| Fusion-P7-LC.14 | CAAGCAGAAGACGGCATACGAGATGTGACTGGAGTTCAGACGTGTGCTCTTCCGATCTNNNNNNNNNNNNACACAGAAGATCTGAATTCAGTGGCACAG |
| Fusion-P7-LC.15 | CAAGCAGAAGACGGCATACGAGATGTGACTGGAGTTCAGACGTGTGCTCTTCCGATCTNNNNNNNNNNNNACACGACCGATCTGAATTCAGTGGCACAG |
| Fusion-P7-LC.16 | CAAGCAGAAGACGGCATACGAGATGTGACTGGAGTTCAGACGTGTGCTCTTCCGATCTNNNNNNNNNNNNACAGATTCGATCTGAATTCAGTGGCACAG |
| Fusion-P7-LC.17 | CAAGCAGAAGACGGCATACGAGATGTGACTGGAGTTCAGACGTGTGCTCTTCCGATCTNNNNNNNNNNNNACAGCAGAGATCTGAATTCAGTGGCACAG |
| Fusion-P7-LC.18 | CAAGCAGAAGACGGCATACGAGATGTGACTGGAGTTCAGACGTGTGCTCTTCCGATCTNNNNNNNNNNNNACATTGGCGATCTGAATTCAGTGGCACAG |
| Fusion-P7-LC.19 | CAAGCAGAAGACGGCATACGAGATGTGACTGGAGTTCAGACGTGTGCTCTTCCGATCTNNNNNNNNNNNNACCACTGTGATCTGAATTCAGTGGCACAG |
| Fusion-P7-LC.20 | CAAGCAGAAGACGGCATACGAGATGTGACTGGAGTTCAGACGTGTGCTCTTCCGATCTNNNNNNNNNNNNACCTCCAAGATCTGAATTCAGTGGCACAG |
| Fusion-P7-LC.21 | CAAGCAGAAGACGGCATACGAGATGTGACTGGAGTTCAGACGTGTGCTCTTCCGATCTNNNNNNNNNNNNACGCTCGAGATCTGAATTCAGTGGCACAG |
| Fusion-P7-LC.22 | CAAGCAGAAGACGGCATACGAGATGTGACTGGAGTTCAGACGTGTGCTCTTCCGATCTNNNNNNNNNNNNACGTATCAGATCTGAATTCAGTGGCACAG |
| Fusion-P7-LC.23 | CAAGCAGAAGACGGCATACGAGATGTGACTGGAGTTCAGACGTGTGCTCTTCCGATCTNNNNNNNNNNNNACTATGCAGATCTGAATTCAGTGGCACAG |
| Fusion-P7-LC.24 | CAAGCAGAAGACGGCATACGAGATGTGACTGGAGTTCAGACGTGTGCTCTTCCGATCTNNNNNNNNNNNNAGAGTCAAGATCTGAATTCAGTGGCACAG |
Supplementary Table 3: LC fusion primers used for Illumina sequencing library preparation

## Slide 5
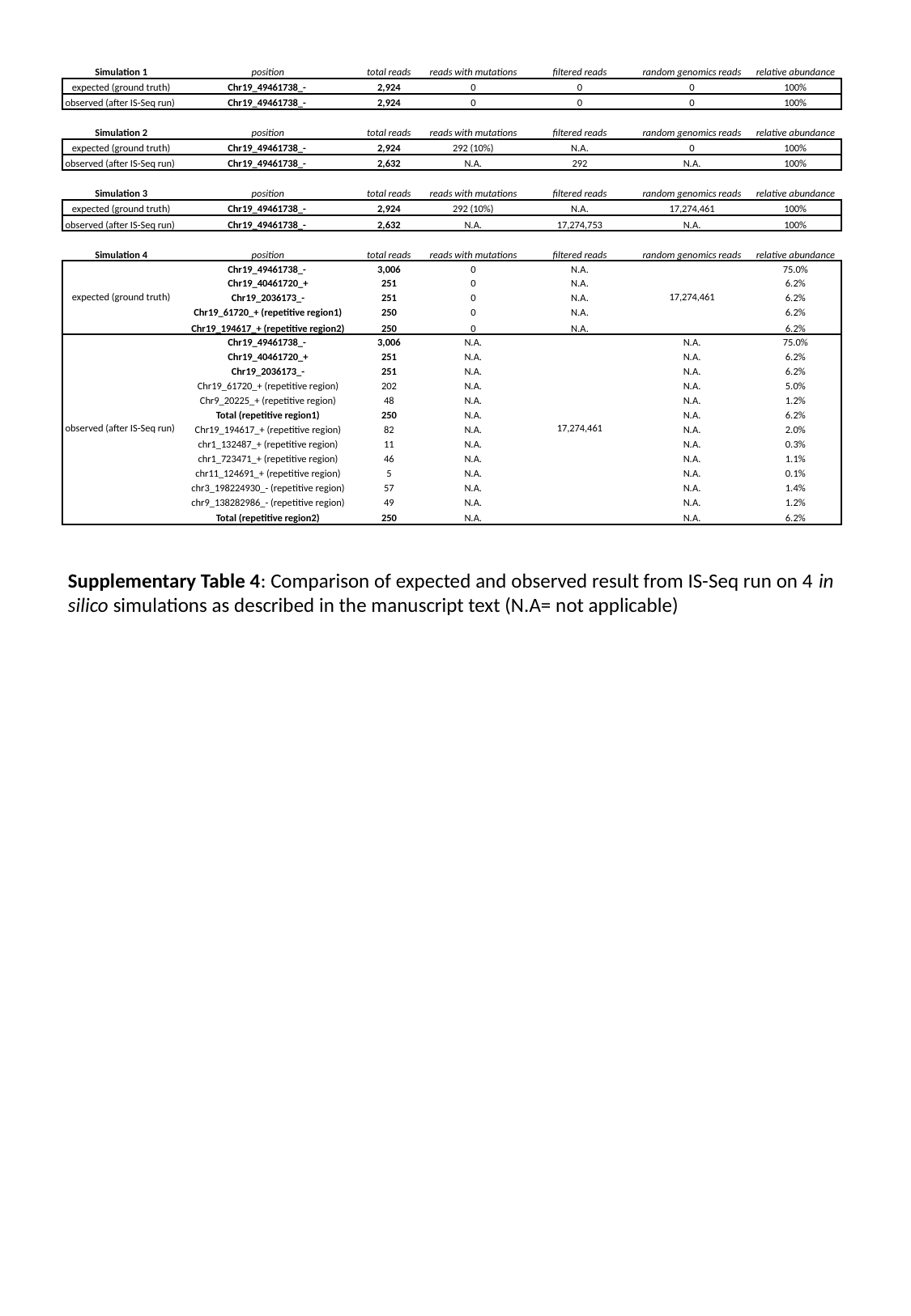

| Simulation 1 | position | total reads | reads with mutations | filtered reads | random genomics reads | relative abundance |
| --- | --- | --- | --- | --- | --- | --- |
| expected (ground truth) | Chr19\_49461738\_- | 2,924 | 0 | 0 | 0 | 100% |
| observed (after IS-Seq run) | Chr19\_49461738\_- | 2,924 | 0 | 0 | 0 | 100% |
| | | | | | | |
| Simulation 2 | position | total reads | reads with mutations | filtered reads | random genomics reads | relative abundance |
| expected (ground truth) | Chr19\_49461738\_- | 2,924 | 292 (10%) | N.A. | 0 | 100% |
| observed (after IS-Seq run) | Chr19\_49461738\_- | 2,632 | N.A. | 292 | N.A. | 100% |
| | | | | | | |
| Simulation 3 | position | total reads | reads with mutations | filtered reads | random genomics reads | relative abundance |
| expected (ground truth) | Chr19\_49461738\_- | 2,924 | 292 (10%) | N.A. | 17,274,461 | 100% |
| observed (after IS-Seq run) | Chr19\_49461738\_- | 2,632 | N.A. | 17,274,753 | N.A. | 100% |
| | | | | | | |
| Simulation 4 | position | total reads | reads with mutations | filtered reads | random genomics reads | relative abundance |
| expected (ground truth) | Chr19\_49461738\_- | 3,006 | 0 | N.A. | 17,274,461 | 75.0% |
| | Chr19\_40461720\_+ | 251 | 0 | N.A. | | 6.2% |
| | Chr19\_2036173\_- | 251 | 0 | N.A. | | 6.2% |
| | Chr19\_61720\_+ (repetitive region1) | 250 | 0 | N.A. | | 6.2% |
| | Chr19\_194617\_+ (repetitive region2) | 250 | 0 | N.A. | | 6.2% |
| observed (after IS-Seq run) | Chr19\_49461738\_- | 3,006 | N.A. | 17,274,461 | N.A. | 75.0% |
| | Chr19\_40461720\_+ | 251 | N.A. | | N.A. | 6.2% |
| | Chr19\_2036173\_- | 251 | N.A. | | N.A. | 6.2% |
| | Chr19\_61720\_+ (repetitive region) | 202 | N.A. | | N.A. | 5.0% |
| | Chr9\_20225\_+ (repetitive region) | 48 | N.A. | | N.A. | 1.2% |
| | Total (repetitive region1) | 250 | N.A. | | N.A. | 6.2% |
| | Chr19\_194617\_+ (repetitive region) | 82 | N.A. | | N.A. | 2.0% |
| | chr1\_132487\_+ (repetitive region) | 11 | N.A. | | N.A. | 0.3% |
| | chr1\_723471\_+ (repetitive region) | 46 | N.A. | | N.A. | 1.1% |
| | chr11\_124691\_+ (repetitive region) | 5 | N.A. | | N.A. | 0.1% |
| | chr3\_198224930\_- (repetitive region) | 57 | N.A. | | N.A. | 1.4% |
| | chr9\_138282986\_- (repetitive region) | 49 | N.A. | | N.A. | 1.2% |
| | Total (repetitive region2) | 250 | N.A. | | N.A. | 6.2% |
Supplementary Table 4: Comparison of expected and observed result from IS-Seq run on 4 in silico simulations as described in the manuscript text (N.A= not applicable)

## Slide 6
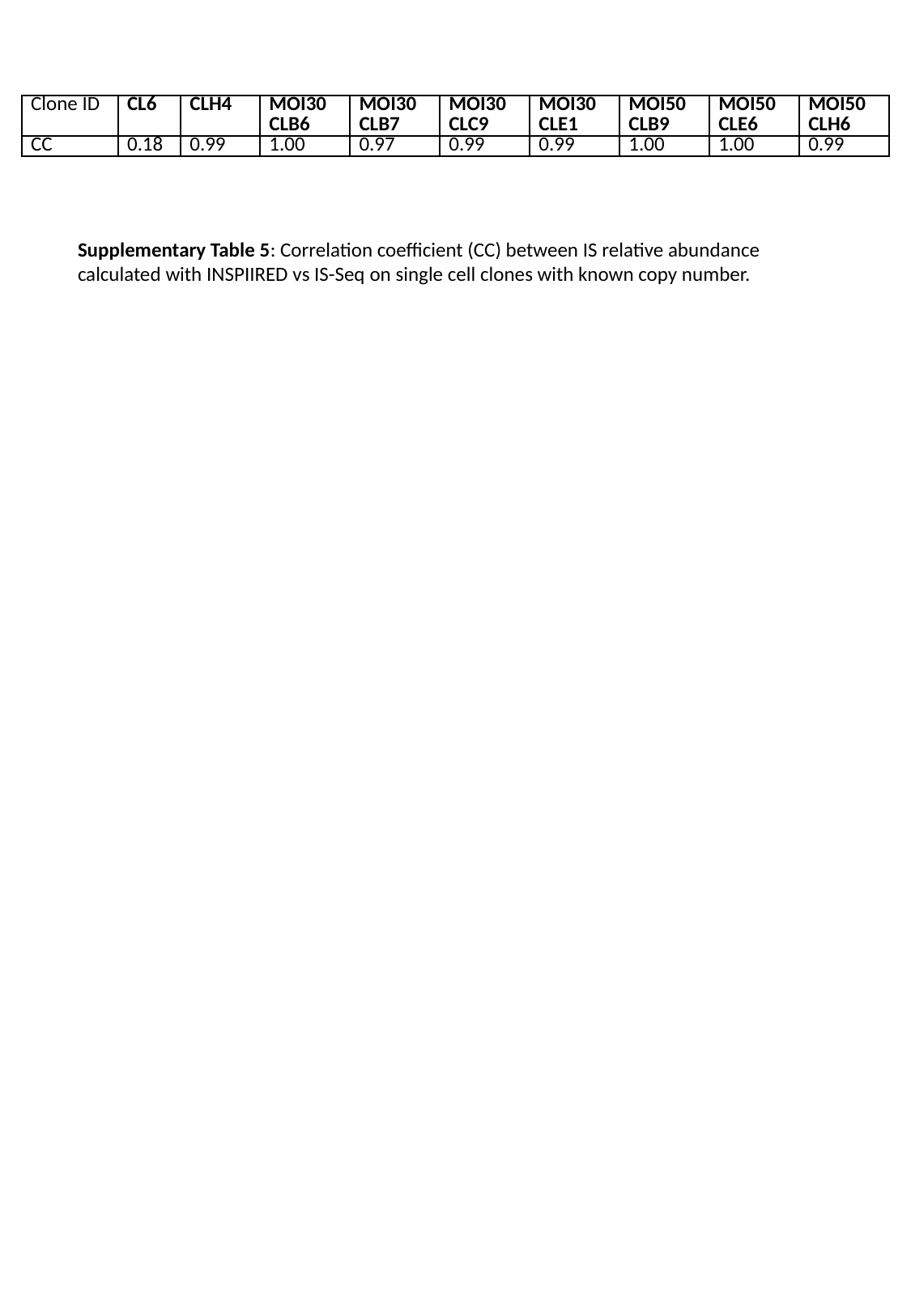

| Clone ID | CL6 | CLH4 | MOI30 CLB6 | MOI30 CLB7 | MOI30 CLC9 | MOI30 CLE1 | MOI50 CLB9 | MOI50 CLE6 | MOI50 CLH6 |
| --- | --- | --- | --- | --- | --- | --- | --- | --- | --- |
| CC | 0.18 | 0.99 | 1.00 | 0.97 | 0.99 | 0.99 | 1.00 | 1.00 | 0.99 |
Supplementary Table 5: Correlation coefficient (CC) between IS relative abundance calculated with INSPIIRED vs IS-Seq on single cell clones with known copy number.

## Slide 7
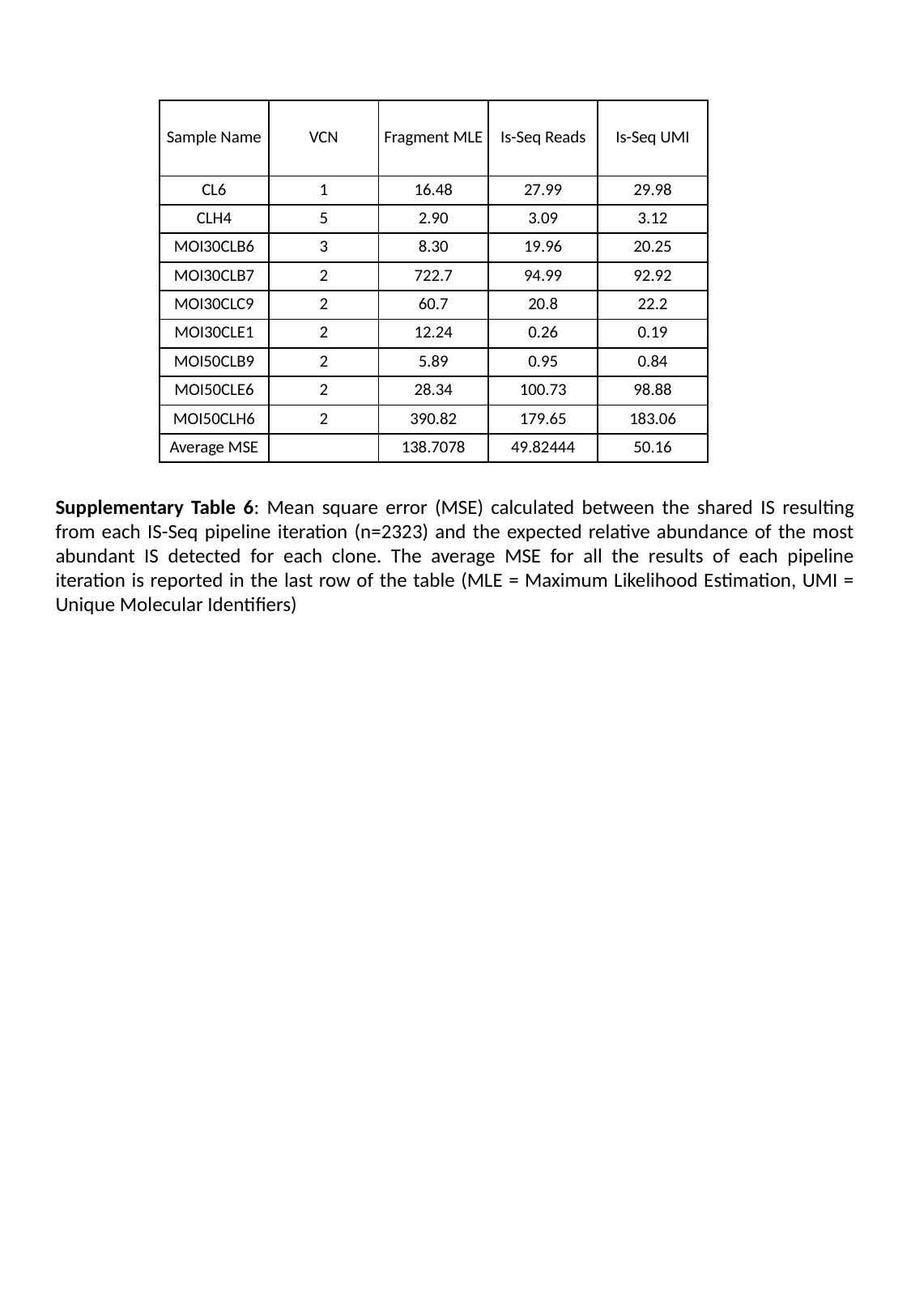

| Sample Name | VCN | Fragment MLE | Is-Seq Reads | Is-Seq UMI |
| --- | --- | --- | --- | --- |
| CL6 | 1 | 16.48 | 27.99 | 29.98 |
| CLH4 | 5 | 2.90 | 3.09 | 3.12 |
| MOI30CLB6 | 3 | 8.30 | 19.96 | 20.25 |
| MOI30CLB7 | 2 | 722.7 | 94.99 | 92.92 |
| MOI30CLC9 | 2 | 60.7 | 20.8 | 22.2 |
| MOI30CLE1 | 2 | 12.24 | 0.26 | 0.19 |
| MOI50CLB9 | 2 | 5.89 | 0.95 | 0.84 |
| MOI50CLE6 | 2 | 28.34 | 100.73 | 98.88 |
| MOI50CLH6 | 2 | 390.82 | 179.65 | 183.06 |
| Average MSE | | 138.7078 | 49.82444 | 50.16 |
Supplementary Table 6: Mean square error (MSE) calculated between the shared IS resulting from each IS-Seq pipeline iteration (n=2323) and the expected relative abundance of the most abundant IS detected for each clone. The average MSE for all the results of each pipeline iteration is reported in the last row of the table (MLE = Maximum Likelihood Estimation, UMI = Unique Molecular Identifiers)

## Slide 8
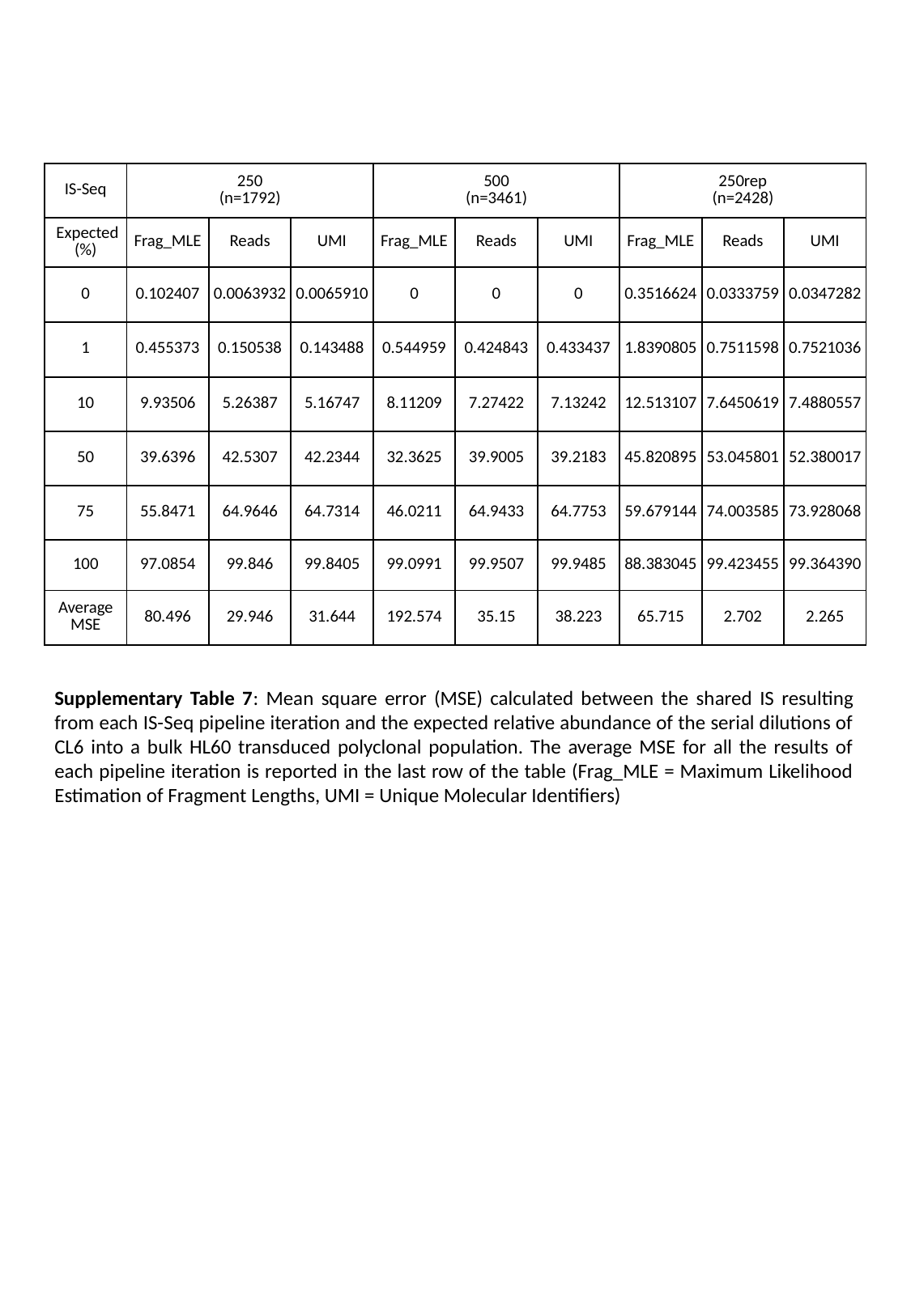

| IS-Seq | 250 (n=1792) | | | 500 (n=3461) | | | 250rep (n=2428) | | |
| --- | --- | --- | --- | --- | --- | --- | --- | --- | --- |
| Expected (%) | Frag\_MLE | Reads | UMI | Frag\_MLE | Reads | UMI | Frag\_MLE | Reads | UMI |
| 0 | 0.102407 | 0.0063932 | 0.0065910 | 0 | 0 | 0 | 0.3516624 | 0.0333759 | 0.0347282 |
| 1 | 0.455373 | 0.150538 | 0.143488 | 0.544959 | 0.424843 | 0.433437 | 1.8390805 | 0.7511598 | 0.7521036 |
| 10 | 9.93506 | 5.26387 | 5.16747 | 8.11209 | 7.27422 | 7.13242 | 12.513107 | 7.6450619 | 7.4880557 |
| 50 | 39.6396 | 42.5307 | 42.2344 | 32.3625 | 39.9005 | 39.2183 | 45.820895 | 53.045801 | 52.380017 |
| 75 | 55.8471 | 64.9646 | 64.7314 | 46.0211 | 64.9433 | 64.7753 | 59.679144 | 74.003585 | 73.928068 |
| 100 | 97.0854 | 99.846 | 99.8405 | 99.0991 | 99.9507 | 99.9485 | 88.383045 | 99.423455 | 99.364390 |
| Average MSE | 80.496 | 29.946 | 31.644 | 192.574 | 35.15 | 38.223 | 65.715 | 2.702 | 2.265 |
Supplementary Table 7: Mean square error (MSE) calculated between the shared IS resulting from each IS-Seq pipeline iteration and the expected relative abundance of the serial dilutions of CL6 into a bulk HL60 transduced polyclonal population. The average MSE for all the results of each pipeline iteration is reported in the last row of the table (Frag_MLE = Maximum Likelihood Estimation of Fragment Lengths, UMI = Unique Molecular Identifiers)

## Slide 9
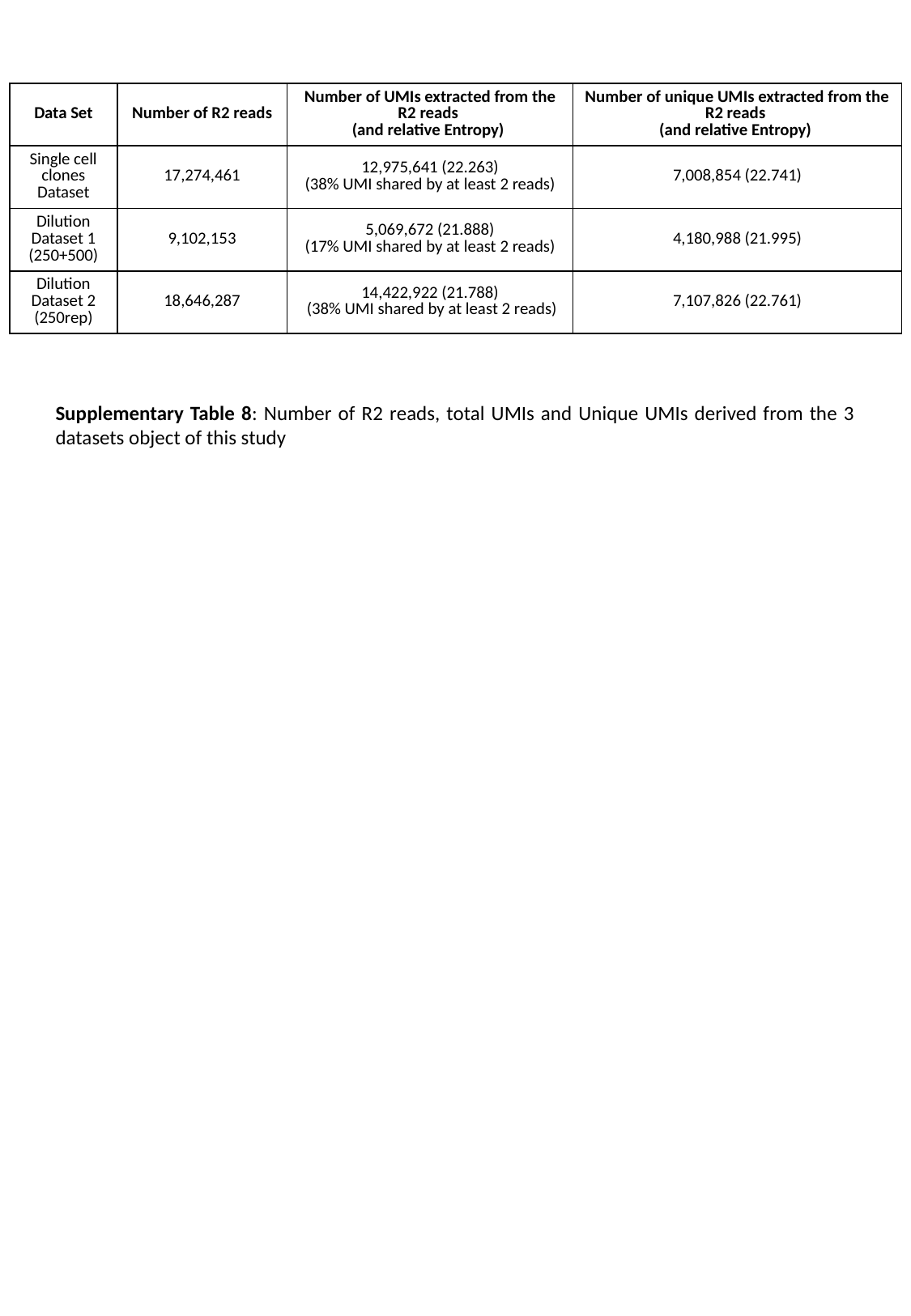

| Data Set | Number of R2 reads | Number of UMIs extracted from the R2 reads (and relative Entropy) | Number of unique UMIs extracted from the R2 reads (and relative Entropy) |
| --- | --- | --- | --- |
| Single cell clones Dataset | 17,274,461 | 12,975,641 (22.263) (38% UMI shared by at least 2 reads) | 7,008,854 (22.741) |
| Dilution Dataset 1 (250+500) | 9,102,153 | 5,069,672 (21.888) (17% UMI shared by at least 2 reads) | 4,180,988 (21.995) |
| Dilution Dataset 2 (250rep) | 18,646,287 | 14,422,922 (21.788) (38% UMI shared by at least 2 reads) | 7,107,826 (22.761) |
Supplementary Table 8: Number of R2 reads, total UMIs and Unique UMIs derived from the 3 datasets object of this study

## Slide 10
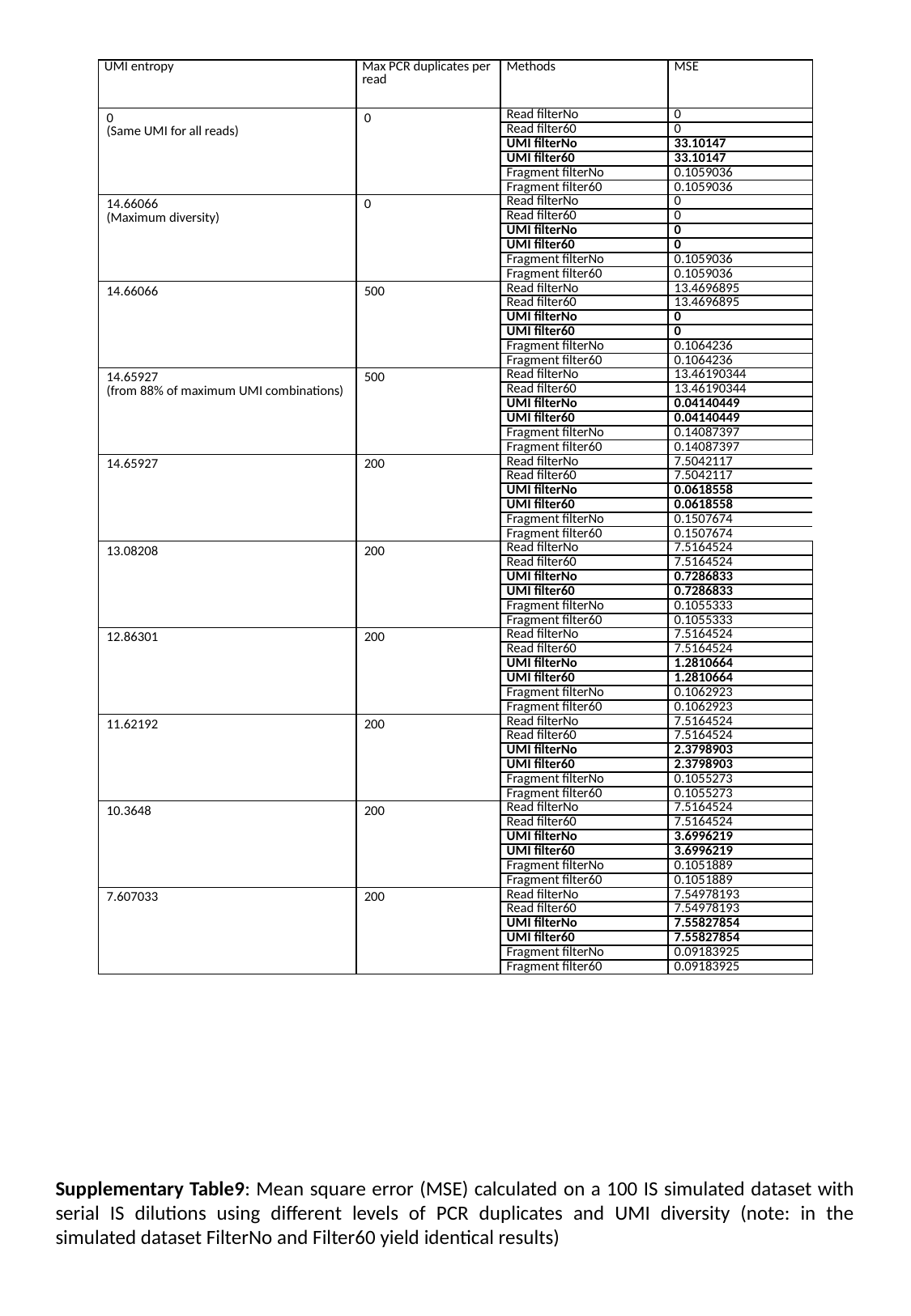

| UMI entropy | Max PCR duplicates per read | Methods | MSE |
| --- | --- | --- | --- |
| 0 (Same UMI for all reads) | 0 | Read filterNo | 0 |
| | | Read filter60 | 0 |
| | | UMI filterNo | 33.10147 |
| | | UMI filter60 | 33.10147 |
| | | Fragment filterNo | 0.1059036 |
| | | Fragment filter60 | 0.1059036 |
| 14.66066 (Maximum diversity) | 0 | Read filterNo | 0 |
| | | Read filter60 | 0 |
| | | UMI filterNo | 0 |
| | | UMI filter60 | 0 |
| | | Fragment filterNo | 0.1059036 |
| | | Fragment filter60 | 0.1059036 |
| 14.66066 | 500 | Read filterNo | 13.4696895 |
| | | Read filter60 | 13.4696895 |
| | | UMI filterNo | 0 |
| | | UMI filter60 | 0 |
| | | Fragment filterNo | 0.1064236 |
| | | Fragment filter60 | 0.1064236 |
| 14.65927 (from 88% of maximum UMI combinations) | 500 | Read filterNo | 13.46190344 |
| | | Read filter60 | 13.46190344 |
| | | UMI filterNo | 0.04140449 |
| | | UMI filter60 | 0.04140449 |
| | | Fragment filterNo | 0.14087397 |
| | | Fragment filter60 | 0.14087397 |
| 14.65927 | 200 | Read filterNo | 7.5042117 |
| | | Read filter60 | 7.5042117 |
| | | UMI filterNo | 0.0618558 |
| | | UMI filter60 | 0.0618558 |
| | | Fragment filterNo | 0.1507674 |
| | | Fragment filter60 | 0.1507674 |
| 13.08208 | 200 | Read filterNo | 7.5164524 |
| | | Read filter60 | 7.5164524 |
| | | UMI filterNo | 0.7286833 |
| | | UMI filter60 | 0.7286833 |
| | | Fragment filterNo | 0.1055333 |
| | | Fragment filter60 | 0.1055333 |
| 12.86301 | 200 | Read filterNo | 7.5164524 |
| | | Read filter60 | 7.5164524 |
| | | UMI filterNo | 1.2810664 |
| | | UMI filter60 | 1.2810664 |
| | | Fragment filterNo | 0.1062923 |
| | | Fragment filter60 | 0.1062923 |
| 11.62192 | 200 | Read filterNo | 7.5164524 |
| | | Read filter60 | 7.5164524 |
| | | UMI filterNo | 2.3798903 |
| | | UMI filter60 | 2.3798903 |
| | | Fragment filterNo | 0.1055273 |
| | | Fragment filter60 | 0.1055273 |
| 10.3648 | 200 | Read filterNo | 7.5164524 |
| | | Read filter60 | 7.5164524 |
| | | UMI filterNo | 3.6996219 |
| | | UMI filter60 | 3.6996219 |
| | | Fragment filterNo | 0.1051889 |
| | | Fragment filter60 | 0.1051889 |
| 7.607033 | 200 | Read filterNo | 7.54978193 |
| | | Read filter60 | 7.54978193 |
| | | UMI filterNo | 7.55827854 |
| | | UMI filter60 | 7.55827854 |
| | | Fragment filterNo | 0.09183925 |
| | | Fragment filter60 | 0.09183925 |
Supplementary Table9: Mean square error (MSE) calculated on a 100 IS simulated dataset with serial IS dilutions using different levels of PCR duplicates and UMI diversity (note: in the simulated dataset FilterNo and Filter60 yield identical results)
