# Supplementary material for: IS-Seq: a bioinformatics pipeline for integration sites analysis with comprehensive abundance quantification methods
Source: BMC Bioinformatics. 2023 Jul 18;24:286. doi: 10.1186/s12859-023-05390-1 (PMC10354991; doi:10.1186/s12859-023-05390-1)
Supplement: Supplementary file 2 — Additional file 2: Fig. S1. Number of integration sites detected in all samples and datasets analyzed in this manuscript using different windows for IS merging. Datasets reported are a combination of the outputs of filterNo and filter60or filter60 only. Each dot in the plot represent a sample, vertical line indicates the 7bp window. Fig. S2. IS-Seq performance metrics based on Precision-Recall curve, ROC curveand correlation between expected abundance and estimated abundanceon simulated datasets with 100and 1000integration sites. Fig. S3. Frequency distribution of distances between INSPIIRED relative abundance readouts and 500 matrixes randomly generated from the IS-Seq relative abundance results. Fig. S4. Relative abundance of insertion sitesdetected in single cell clones using different pipelines and sequence similarity thresholdsbased on the Maximum Likelihood Estimate of fragment lengths.. Fig. S5. A) Relative abundance of MLE of fragment lenghts of IS collected from CLB7, CLH6 and CL6 using INSPIIRED combining unique hits and sequence clusters from the multihits at different sequence similarity thresholds. Fig. S6. A) Relative abundance of MLE of fragment lenghts of IS collected from CLB7 using INSPIIRED unique, multihits and a combination thereof at different sequence similarity thresholds. B) Absolute abundance of the 2nd top IS detected in CLB7 using different SST and different INSPIIRED functions. Fig. S7. Sharing of identical insertions detected with INSPIIRED and IS-Seq in the HL60 polyclonal bulk population. Fig. S8. Left panels show the distribution of the top 100 most abundant UMIs reads counts observed in the 3 datasets object of this study. Each bar represents an individual UMI whose sequence is reported in the x-axis. Below each plot is reported the entropy value of the whole UMI population. The center panels show the same analysis performed on 100 a random subsamplingof UMIs as described in the main text. Range of entropy values is reported below e [file 12859_2023_5390_MOESM2_ESM.pptx]

## Slide 1
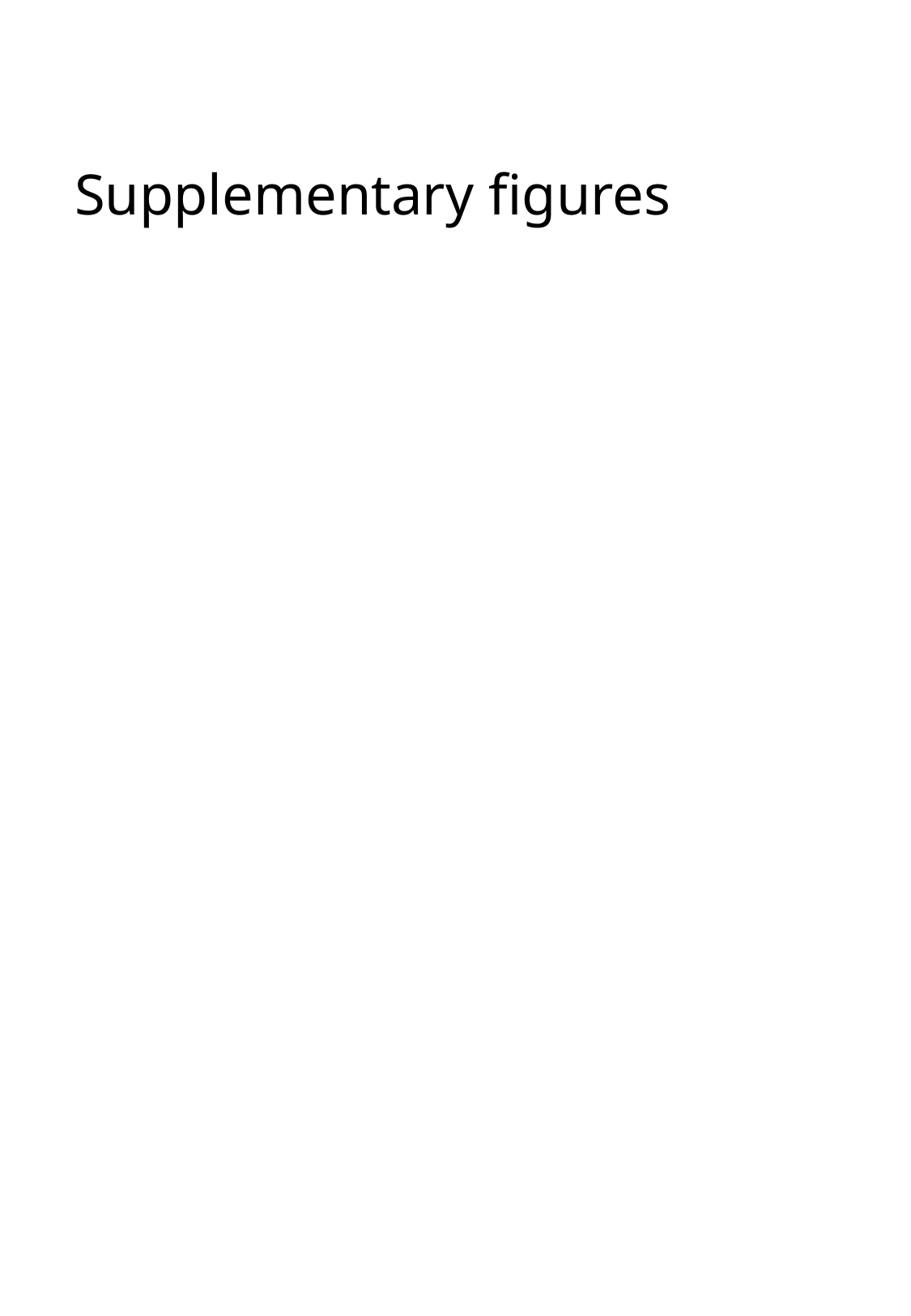

# Supplementary figures

## Slide 2
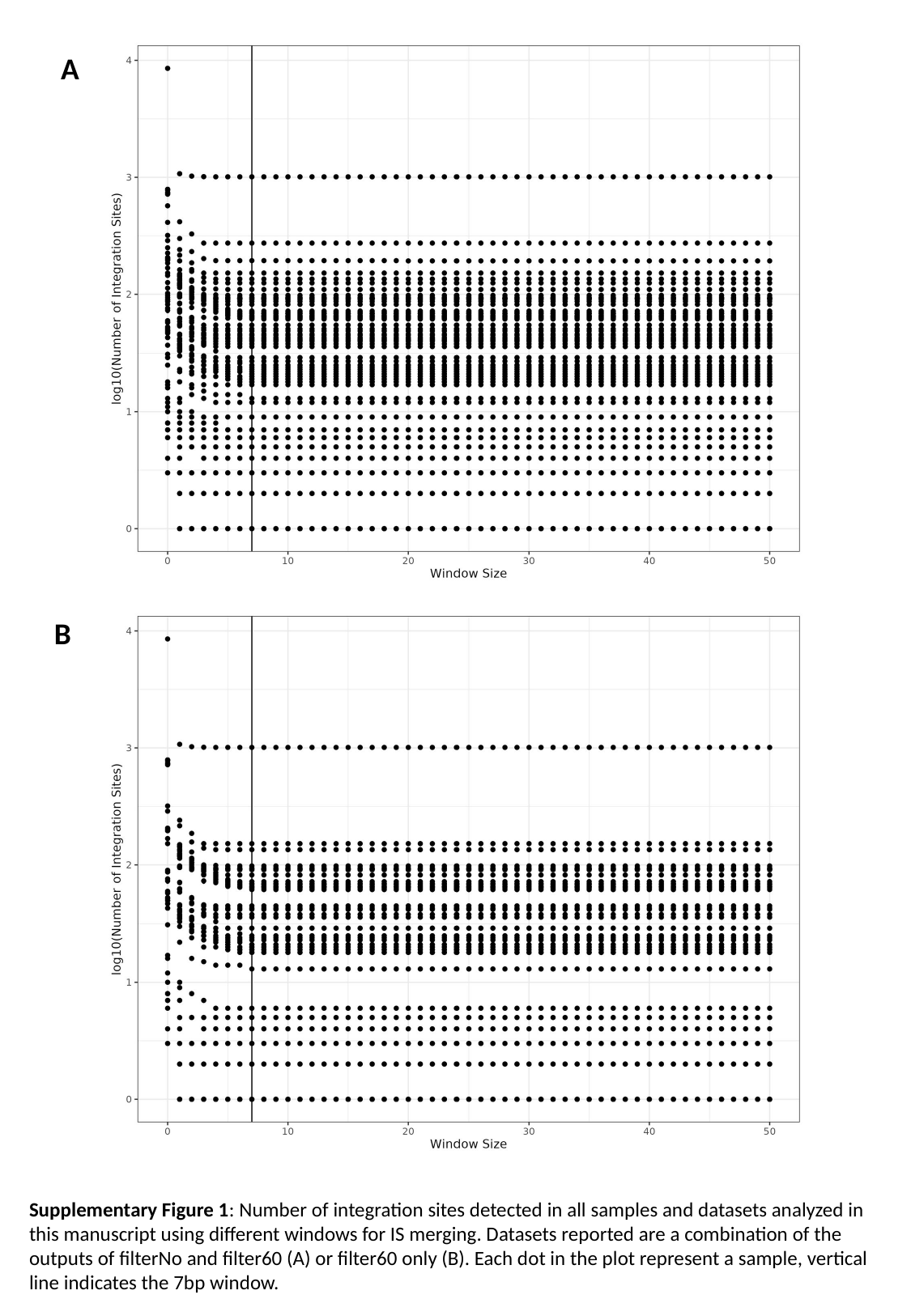

A
B
Supplementary Figure 1: Number of integration sites detected in all samples and datasets analyzed in this manuscript using different windows for IS merging. Datasets reported are a combination of the outputs of filterNo and filter60 (A) or filter60 only (B). Each dot in the plot represent a sample, vertical line indicates the 7bp window.

## Slide 3
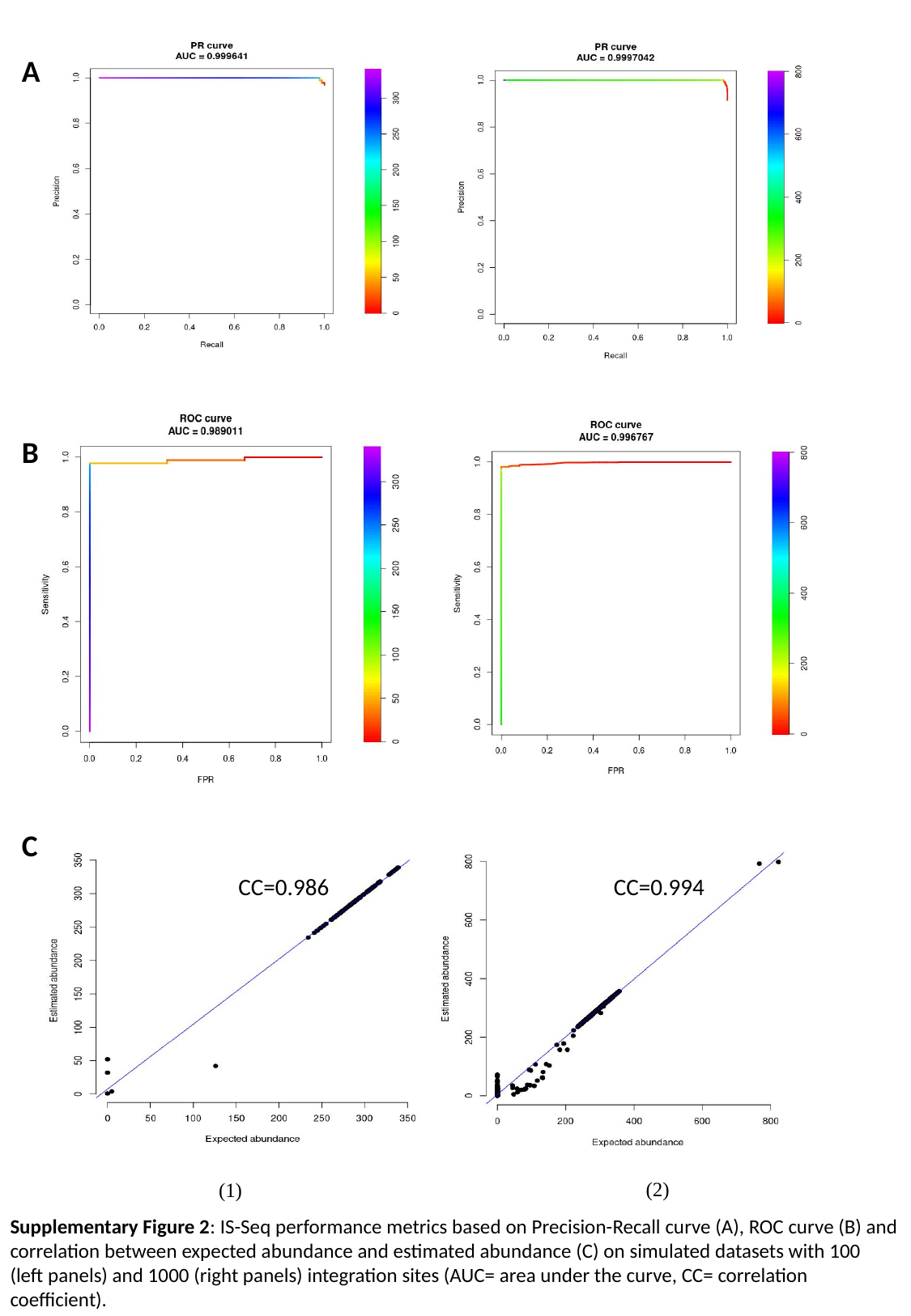

A
B
AUC=0.997
AUC=0.989
C
CC=0.986
CC=0.994
(2)
(1)
Supplementary Figure 2: IS-Seq performance metrics based on Precision-Recall curve (A), ROC curve (B) and correlation between expected abundance and estimated abundance (C) on simulated datasets with 100 (left panels) and 1000 (right panels) integration sites (AUC= area under the curve, CC= correlation coefficient).

## Slide 4
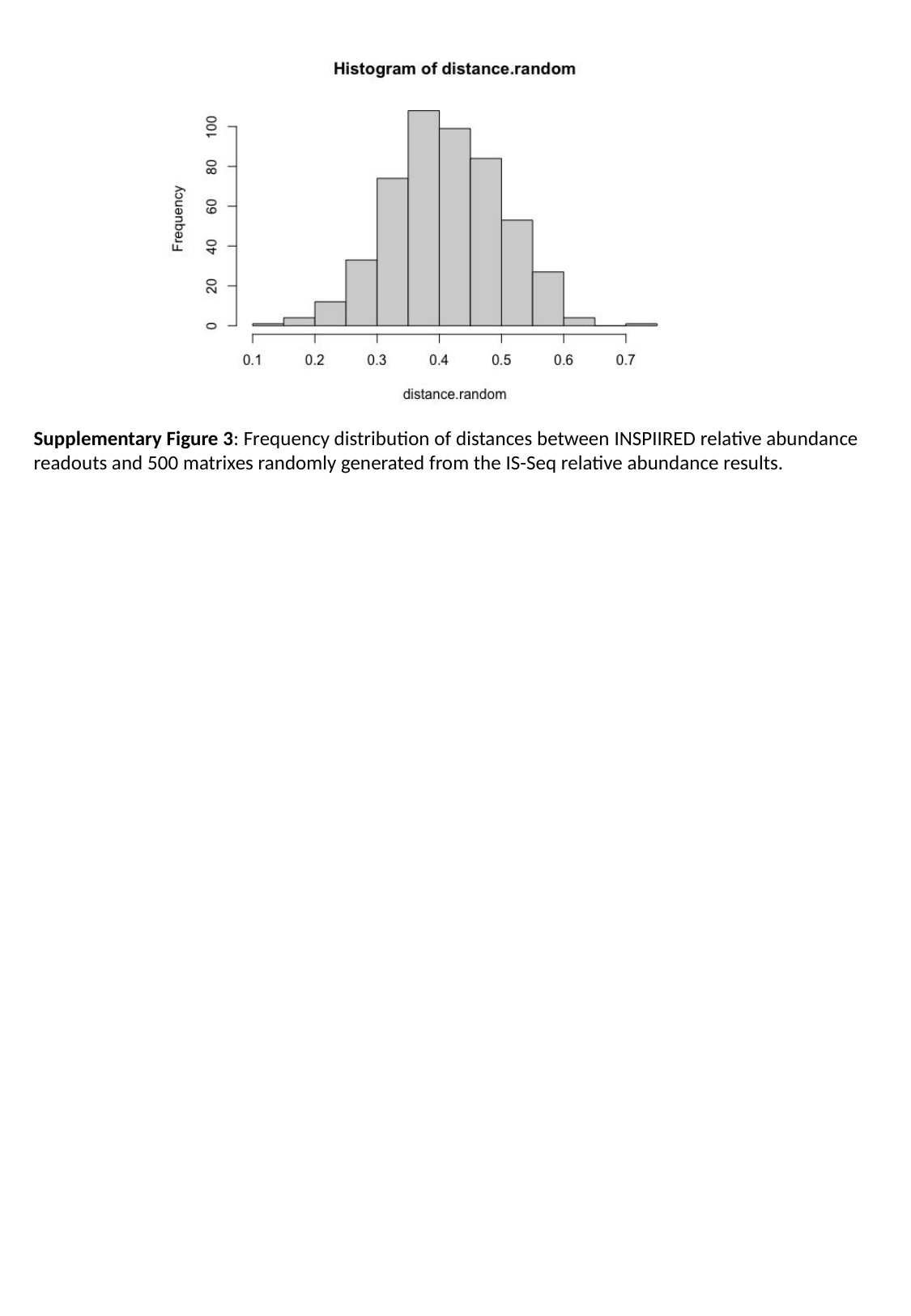

Supplementary Figure 3: Frequency distribution of distances between INSPIIRED relative abundance readouts and 500 matrixes randomly generated from the IS-Seq relative abundance results.

## Slide 5
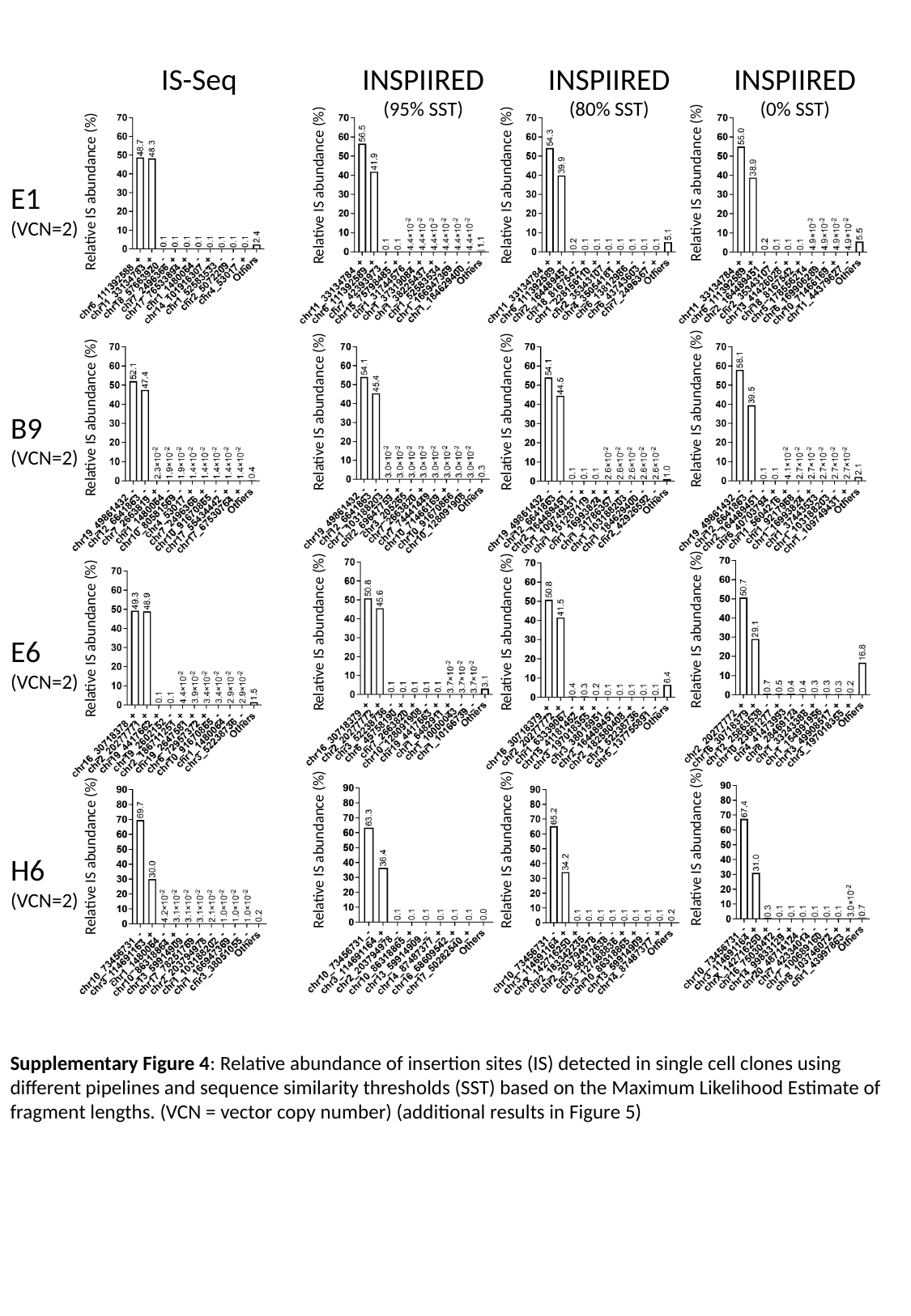

INSPIIRED
(95% SST)
INSPIIRED
(80% SST)
INSPIIRED
(0% SST)
IS-Seq
Relative IS abundance (%)
Relative IS abundance (%)
Relative IS abundance (%)
E1
(VCN=2)
Relative IS abundance (%)
Relative IS abundance (%)
Relative IS abundance (%)
Relative IS abundance (%)
B9
(VCN=2)
Relative IS abundance (%)
Relative IS abundance (%)
Relative IS abundance (%)
Relative IS abundance (%)
Relative IS abundance (%)
E6
(VCN=2)
Relative IS abundance (%)
Relative IS abundance (%)
Relative IS abundance (%)
Relative IS abundance (%)
H6
(VCN=2)
Supplementary Figure 4: Relative abundance of insertion sites (IS) detected in single cell clones using different pipelines and sequence similarity thresholds (SST) based on the Maximum Likelihood Estimate of fragment lengths. (VCN = vector copy number) (additional results in Figure 5)

## Slide 6
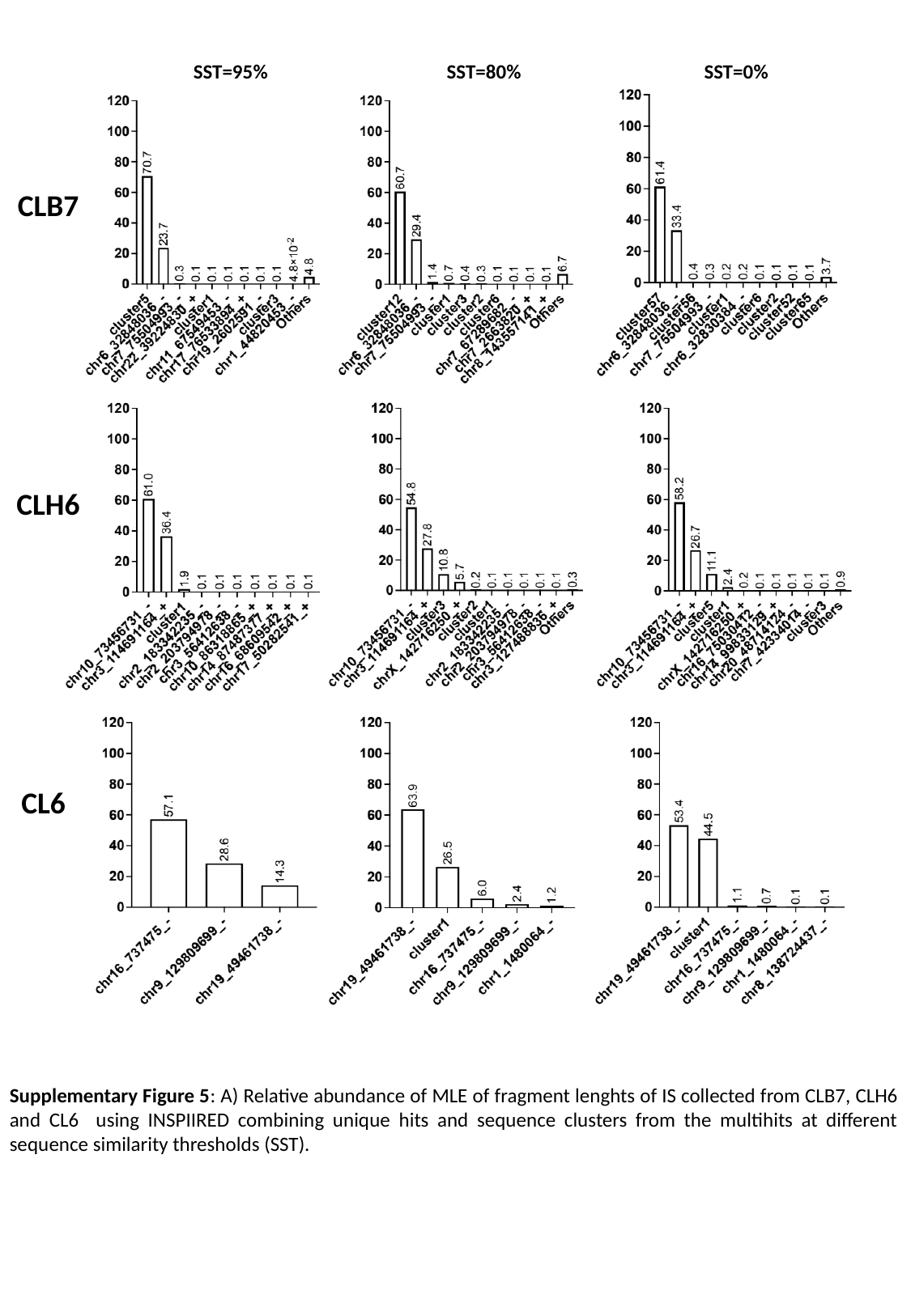

SST=0%
SST=80%
SST=95%
CLB7
CLH6
CL6
Supplementary Figure 5: A) Relative abundance of MLE of fragment lenghts of IS collected from CLB7, CLH6 and CL6 using INSPIIRED combining unique hits and sequence clusters from the multihits at different sequence similarity thresholds (SST).

## Slide 7
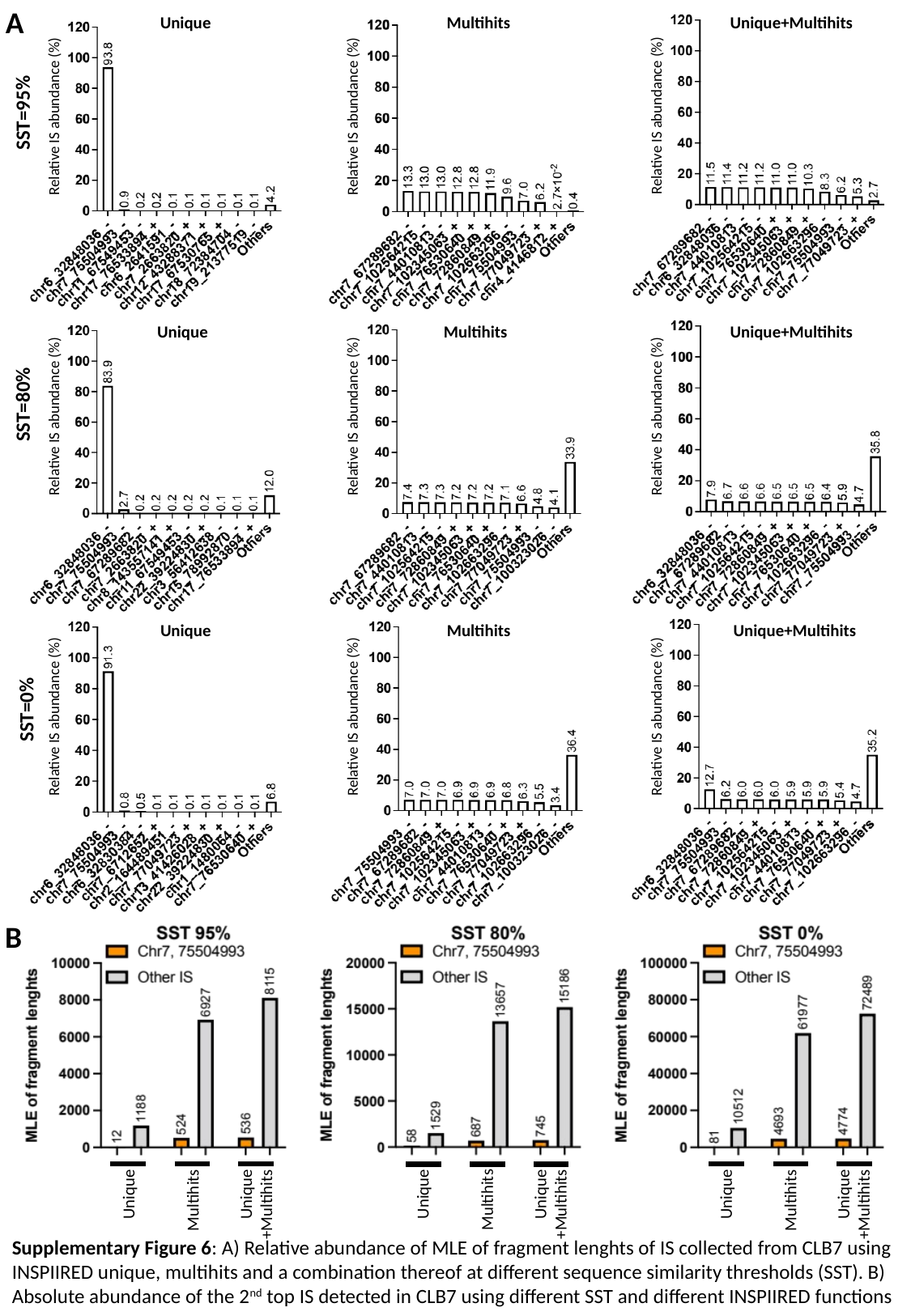

A
Unique
Multihits
Unique+Multihits
Relative IS abundance (%)
Relative IS abundance (%)
Relative IS abundance (%)
Unique
Multihits
Unique+Multihits
Relative IS abundance (%)
Relative IS abundance (%)
Relative IS abundance (%)
Unique
Multihits
Unique+Multihits
Relative IS abundance (%)
Relative IS abundance (%)
Relative IS abundance (%)
SST=95%
SST=80%
SST=0%
B
Unique
+Multihits
Unique
Unique
+Multihits
Unique
Unique
+Multihits
Unique
Multihits
Multihits
Multihits
Supplementary Figure 6: A) Relative abundance of MLE of fragment lenghts of IS collected from CLB7 using INSPIIRED unique, multihits and a combination thereof at different sequence similarity thresholds (SST). B) Absolute abundance of the 2nd top IS detected in CLB7 using different SST and different INSPIIRED functions

## Slide 8
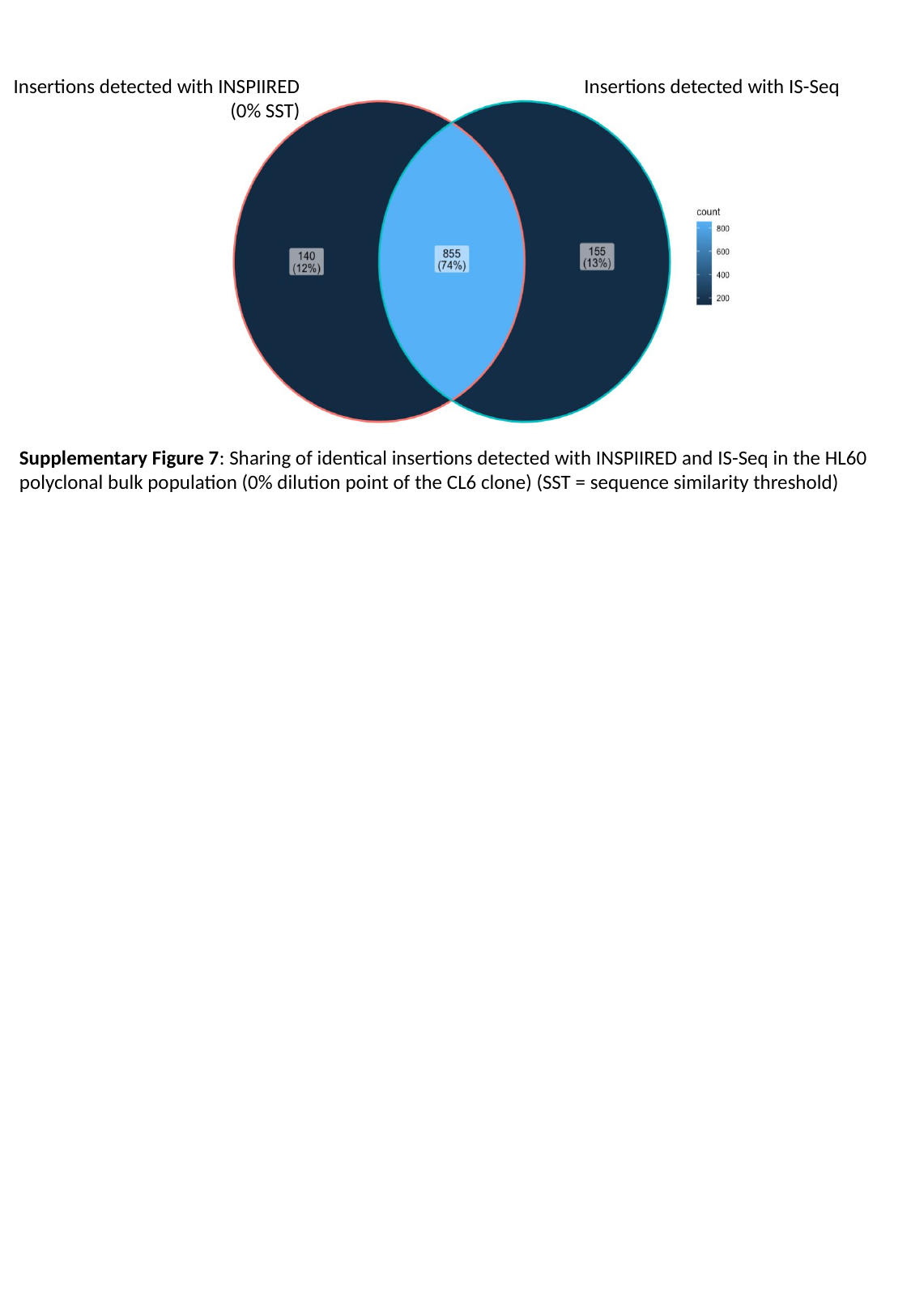

Insertions detected with INSPIIRED
(0% SST)
Insertions detected with IS-Seq
Supplementary Figure 7: Sharing of identical insertions detected with INSPIIRED and IS-Seq in the HL60 polyclonal bulk population (0% dilution point of the CL6 clone) (SST = sequence similarity threshold)

## Slide 9
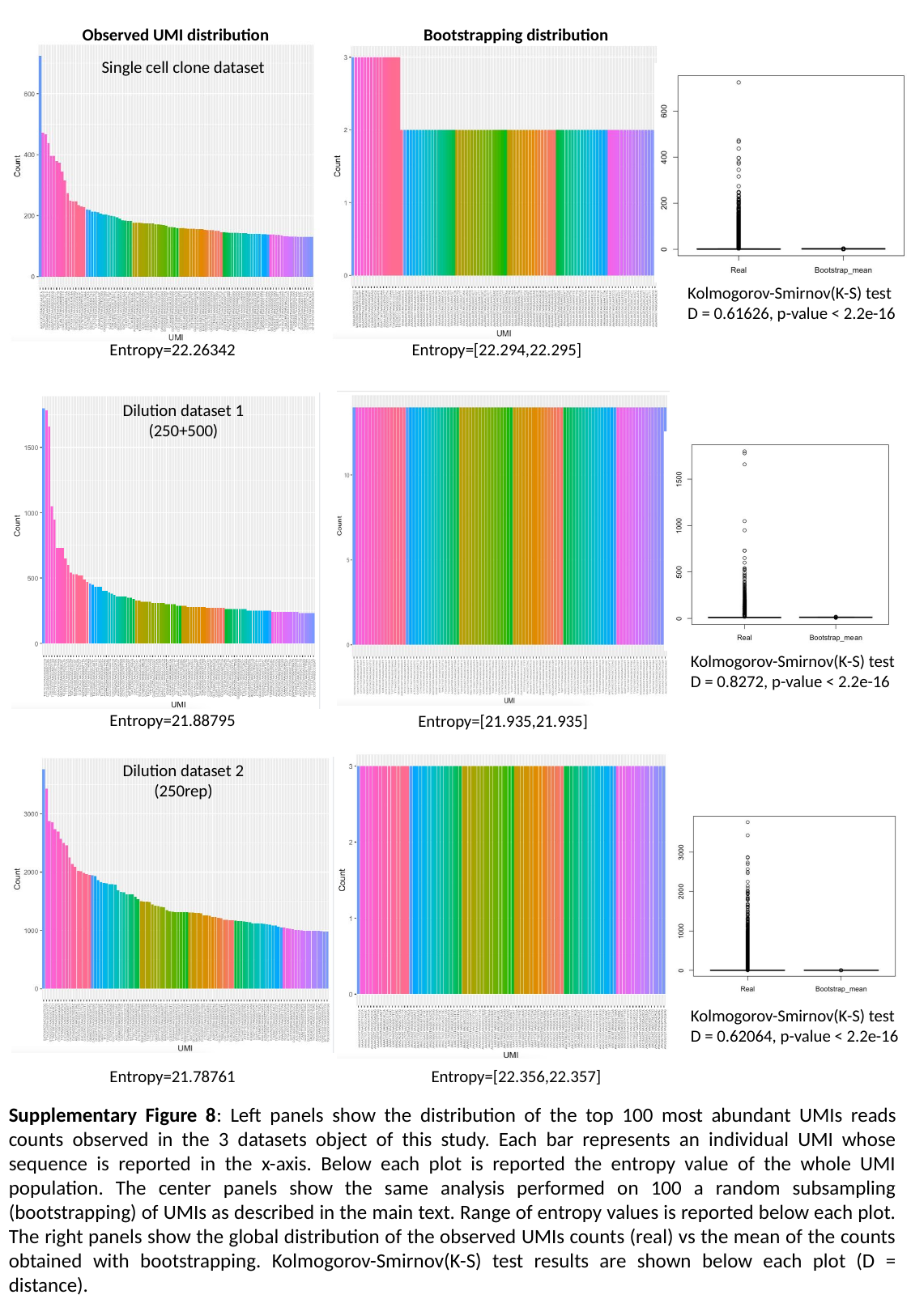

Bootstrapping distribution
Observed UMI distribution
Single cell clone dataset
Kolmogorov-Smirnov(K-S) test
D = 0.61626, p-value < 2.2e-16
Entropy=[22.294,22.295]
Entropy=22.26342
Dilution dataset 1
(250+500)
Kolmogorov-Smirnov(K-S) test
D = 0.8272, p-value < 2.2e-16
Entropy=21.88795
Entropy=[21.935,21.935]
Dilution dataset 2
(250rep)
Kolmogorov-Smirnov(K-S) test
D = 0.62064, p-value < 2.2e-16
Entropy=21.78761
Entropy=[22.356,22.357]
Supplementary Figure 8: Left panels show the distribution of the top 100 most abundant UMIs reads counts observed in the 3 datasets object of this study. Each bar represents an individual UMI whose sequence is reported in the x-axis. Below each plot is reported the entropy value of the whole UMI population. The center panels show the same analysis performed on 100 a random subsampling (bootstrapping) of UMIs as described in the main text. Range of entropy values is reported below each plot. The right panels show the global distribution of the observed UMIs counts (real) vs the mean of the counts obtained with bootstrapping. Kolmogorov-Smirnov(K-S) test results are shown below each plot (D = distance).

## Slide 10
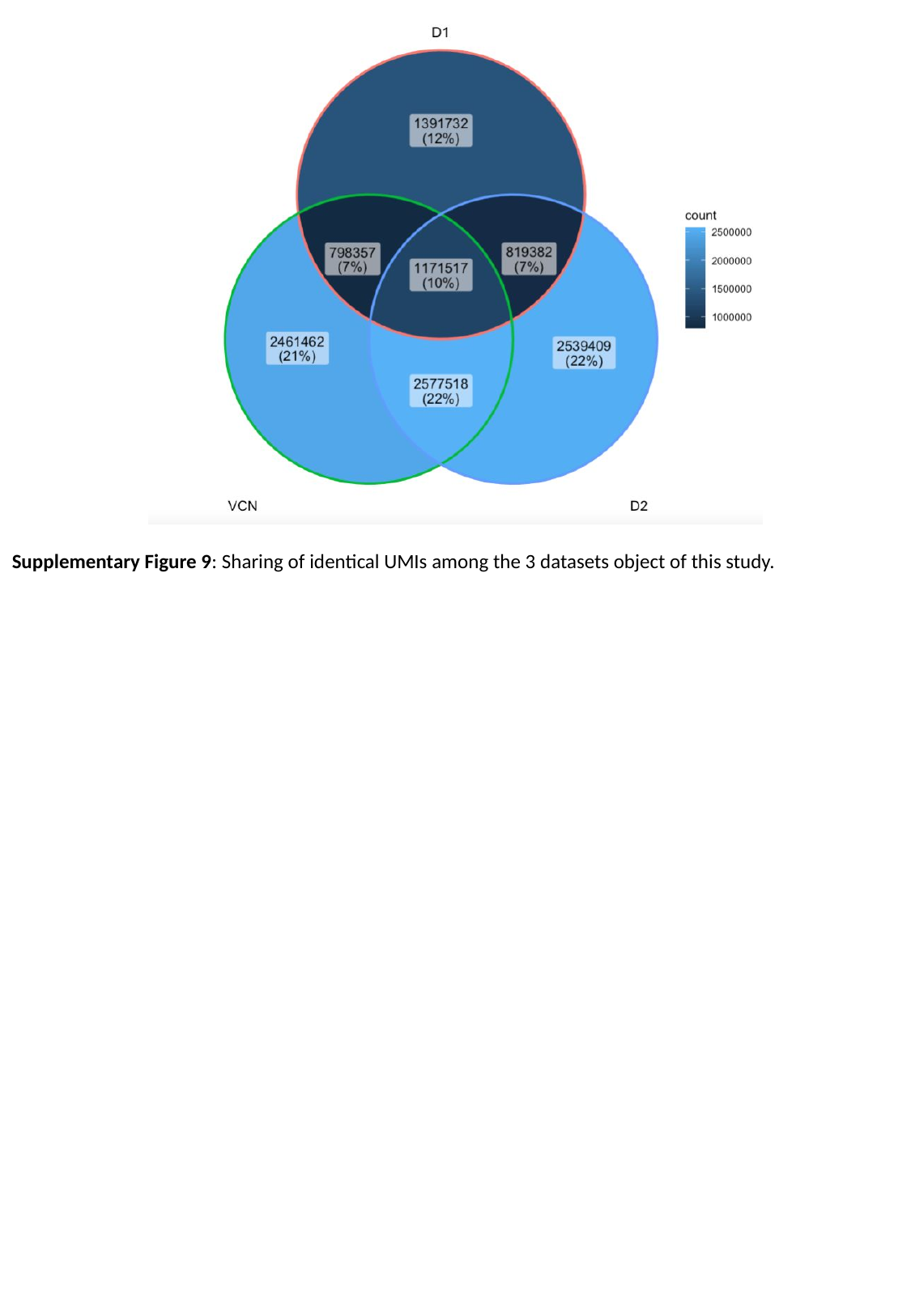

Supplementary Figure 9: Sharing of identical UMIs among the 3 datasets object of this study.

## Slide 11
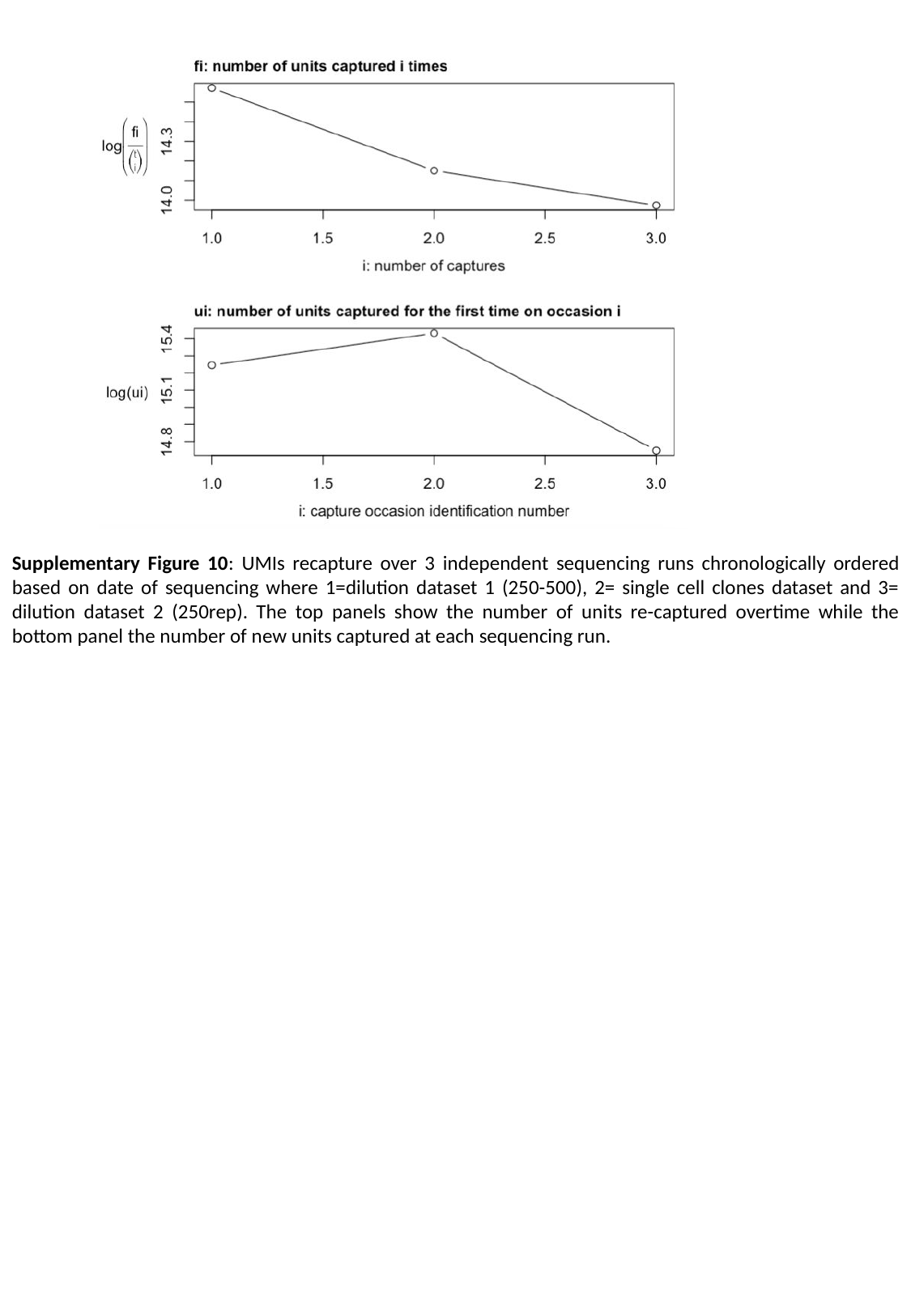

Supplementary Figure 10: UMIs recapture over 3 independent sequencing runs chronologically ordered based on date of sequencing where 1=dilution dataset 1 (250-500), 2= single cell clones dataset and 3= dilution dataset 2 (250rep). The top panels show the number of units re-captured overtime while the bottom panel the number of new units captured at each sequencing run.

## Slide 12
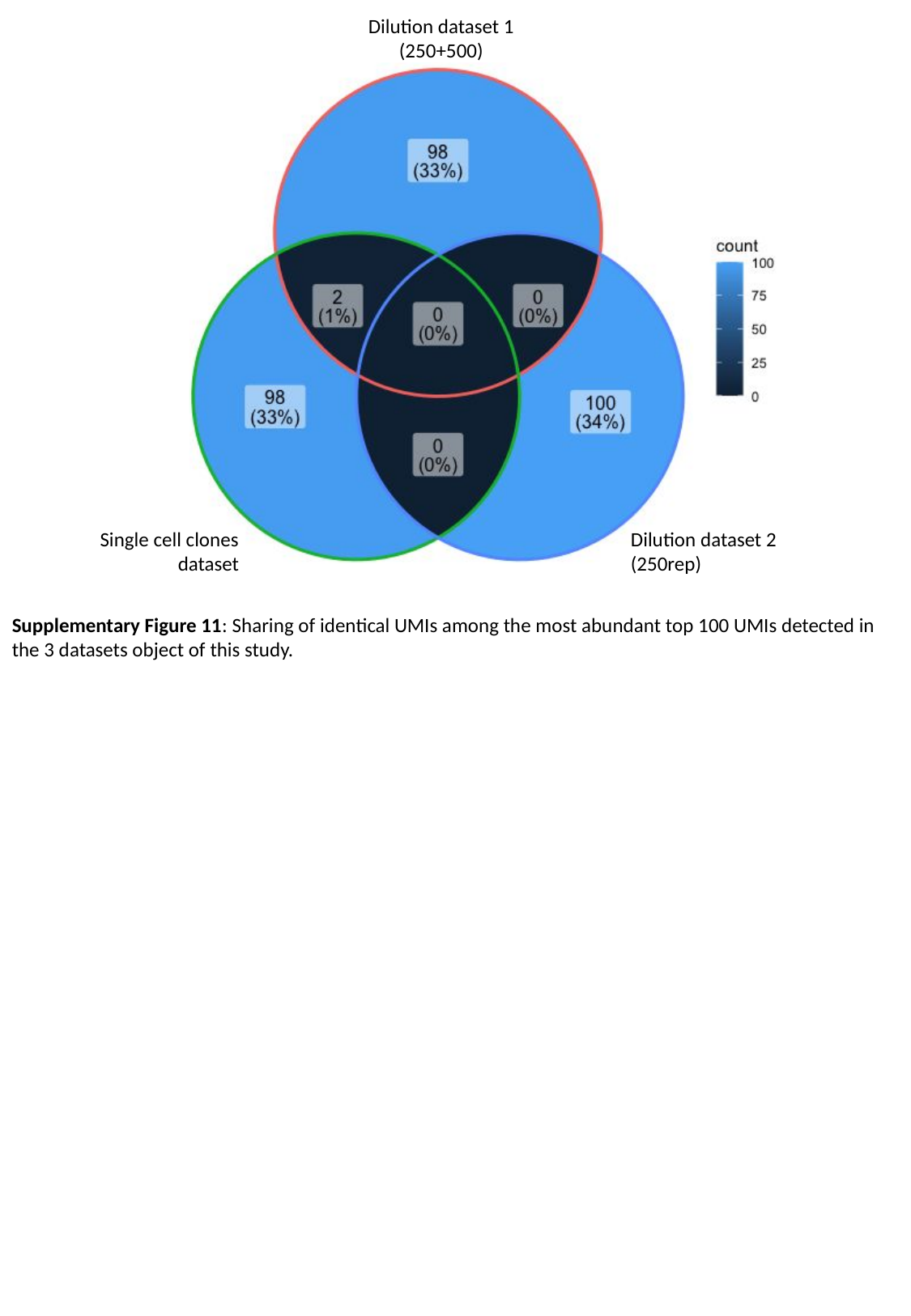

Dilution dataset 1
(250+500)
Single cell clones
dataset
Dilution dataset 2
(250rep)
Supplementary Figure 11: Sharing of identical UMIs among the most abundant top 100 UMIs detected in the 3 datasets object of this study.
